# Stabilization of propene molybdenum and tungsten half–sandwich complexes by intramolecular coordination of a thioether function†

**DOI:** 10.1039/d3ra03383j

**Published:** 2023-06-30

**Authors:** Lukáš Hanzl, Jaromír Vinklárek, Libor Dostál, Ivana Císařová, Miroslava Litecká, Jan Honzíček

**Affiliations:** a Department of General and Inorganic Chemistry, Faculty of Chemical Technology, University of Pardubice Studentská 573 532 10 Pardubice Czech Republic; b Department of Inorganic Chemistry, Faculty of Science, Charles University in Prague Hlavova 2030/8 128 43 Prague 2 Czech Republic; c Department of Materials Chemistry, Institute of Inorganic Chemistry of the CAS Husinec-Řež 1001 25068 Řež Czech Republic; d Institute of Chemistry and Technology of Macromolecular Materials, Faculty of Chemical Technology, University of Pardubice Studentská 573 532 10 Pardubice Czech Republic jan.honzicek@upce.cz

## Abstract

This study reports the stabilizing effect of an intramolecularly coordinated thioether function in propene complexes of the general formula [{η^5^:κ*S*-C_5_H_4_(CH_2_)_2_SR}M(CO)_2_(η^2^-C_2_H_3_Me)][BF_4_] (M = Mo, W; R = Et, Ph). They are formed by protonation of allyl analogues [{η^5^-C_5_H_4_(CH_2_)_2_SR}M(CO)_2_(η^3^-C_3_H_5_)] by tetrafluoroboric acid in non-coordinating solvents. In contrast to analogues with unsubstituted Cp ligands, these propene complexes are isolable in a pure form and characterized by NMR spectroscopy. The molybdenum compounds are stable at low temperature and the propene ligand can easily be exchanged by thioethers or acetonitrile. Several representatives of the reaction products were characterized by X-ray structure analysis. The stabilization effect in tungsten complexes [{η^5^:κ*S*-C_5_H_4_(CH_2_)_2_SR}W(CO)_2_(η^2^-C_2_H_3_Me)][BF_4_] (R = Et, Ph) was unusually high. The compounds are long-term stable at room temperature and do not undergo ligand exchange reactions even with strong chelators such as 1,10-phenanthroline. The molecular structure of the tungsten propene complex was confirmed by X-ray diffraction analysis on a single crystal.

## Abstract 2

The stabilizing effect of an intramolecularly coordinated thioether function in propene complexes of molybdenum and tungsten is reported.

## Graphical abstract

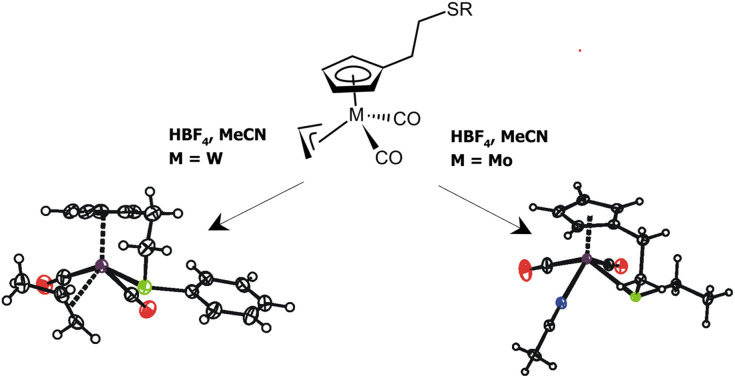

## Introduction

The ability of transition metals to form coordination compounds with alkenes was discovered almost two hundred years ago when W. C. Zeise^1^ synthesized the first compound bearing π-bonded ethylene, K[PtCl_3_(η^2^-C_2_H_4_)]·H_2_O, currently known as Zeise's salt.^1,2^ After elucidation of the alkene coordination mode in the 1950s,^1,3^ a variety of complexes with π-coordinated alkenes have been described, which was motivated by their key role in catalytic olefin oligomerization and polymerization reactions.^4–6^ For example, the specific design of π-alkene intermediates permits the living polymerization catalysis of ethylene at unusually high temperatures to afford ultra-high molecular weight polyethylene with low dispersity.^7^ The effects of supporting ligands on the stability of intermediate π-alkene species are further exemplified in recent studies dealing with ethylene oligomerization.^8,9^

Alkene intermediates are involved in metathesis reactions (*e.g.*, ring-closing metathesis, cross-metathesis, and metathesis polymerization).^10^ These processes are commonly catalyzed by high-valent molybdenum and tungsten complexes^11^ and by ruthenium compounds.^10,12^ The potential of iron catalysts is currently under comprehensive investigation.^13–15^ Gold(i)-ethylene complexes, stabilized with bulky phosphine ligands were investigated as catalysts for the hydroamination of ethylene.^16,17^ Stereoregularity of transition metal catalyzed alkene hydroformylation is controlled upon transfer of hydride ligand to the coordinated alkene. Formed branched aldehydes serve as attractive synthetic precursors for pharmaceuticals.^18^

**Scheme 1:**
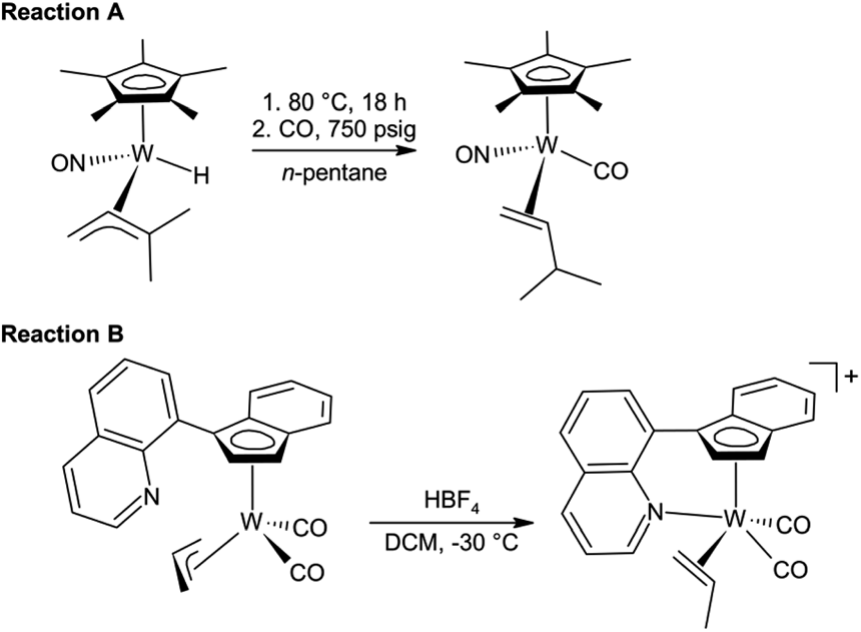
Stabilization of η^2^-alkene tungsten complexes by (a) CO coordination.^19^ (b) Intramolecular coordination.^21^

Alkene intermediates are formed upon C–H activation of hydrocarbons by cyclopentadienyl tungsten complexes [(η^5^-Cp*)W(NO)(R)(η^3^-allyl)] (Cp* = C_5_Me_5_; R = H, neopentyl), as documented on adducts bearing trapped carbon monoxide [(η^5^-Cp*)W(NO)(CO)(η^2^-alkene)] (Scheme 1, reaction A).^19,20^

Recently, we have proven formation of η^2^-propene intermediates upon protonation of η^3^-allyl molybdenum and tungsten compounds [(η^5^-Ind′)M(CO)_2_(η^3^-allyl)] (Ind’ = substituted indenyl; M = Mo, W) by strong acid in non-coordinating solvents. The η^2^-propene intermediates were stabilized by intramolecular coordination of 1-(quinol-8-yl)indenyl ligand. A tungsten complex, presented in Scheme 1 (reaction B), was found to be stable up to 0 °C.^21^ This study further documented that the η^2^-propene ligand, in this type of complex, can be easily exchanged by labile ligands (*e.g.*, dimethyl sulfide) while stronger ligands (*e.g.*, acetonitrile) induce η^3^-to-η^5^-indenyl ring slippage.^21^

The aim of this work is to describe stabilizing effects of the intramolecular coordination on cyclopentadienyl molybdenum and tungsten complexes without an annulated benzene ring. For this purpose, the thioether moiety in the side chain was chosen due to expected hemilabile coordination to the metal. Our secondary aim involves reactivity of η^2^-propene complexes, prepared *in situ*, with thioethers and acetonitrile, as both ligands traditionally form labile complexes with early transition metals.

## Results and discussion

**Scheme 2:**
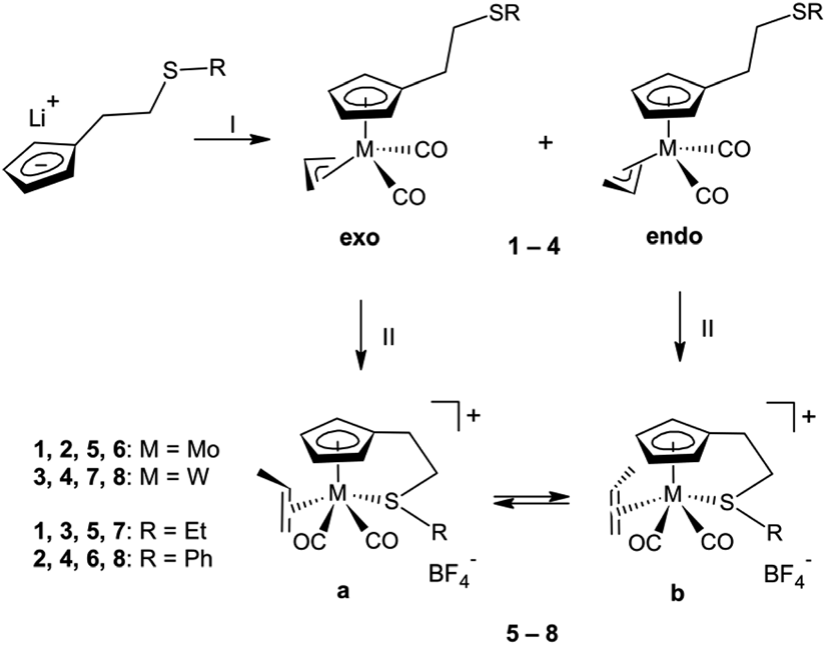
Synthesis of the precursors 1–4 and their protonation in a non-coordinating solvent: (I) [(η^3^-C_3_H_5_)(MeCN)_2_M(CO)_2_Cl] (M = Mo, W); (II) HBF_4_·Et_2_O, CH_2_Cl_2_, −40 °C.

The allyl(cyclopentadienyl) complexes 1–4 were prepared by the reaction of [(η^3^-C_3_H_5_)(MeCN)_2_M(CO)_2_Cl] (M = Mo, W) with lithium cyclopentadienides bearing a thioether group in the side chain LiC_5_H_4_(CH_2_)_2_SR (R = Et, Ph), see Scheme 2. These precursors of propene complexes were isolated and characterized using ^1^H NMR and IR spectroscopy.

^1^H spectra of the complexes 1–4 contained two sets of signals for the allyl ligand, expected for complexes of the [(η^5^-Cp′)M(CO)_2_(η^3^-C_3_H_5_)],^22^ due to the presence of the *exo*/*endo* isomerism of the allyl ligand (Scheme 2). At room temperature, *exo-* and *endo*-conformers appear in molar ratio 7 : 2 for 1 and 2, 5 : 2 for 3 and 2 : 1 for 4. The assignment allyl resonances in 1–4 was aided by data from previously prepared analogues with unsubstituted cyclopentadienyl ligands.^23^ The infrared spectra of 1–4 exhibit two absorption bands in the region of 2000–1800 cm^−1^, assigned to the asymmetric and symmetric stretching modes of the terminal carbonyl ligands (Table 1).

**Table 1:** Wavenumbers (in cm^−1^) of characteristic infrared bands

Compound	*ν*_a_(CO)	*ν*_s_(CO)	*ν*(BF)
1	1935	1850	—
2	1936	1850	—
3	1929	1840	—
4	1929	1839	—
7	2025	1946	1034
8	2025	1915	1026
9	1975	1883	1019
10	1974	1882	1048
11	1986	1885	1026
12	1978	1890	1022
13	1975	1890	1022
14	1984	1901	1030
15	1982	1888	1029
16	1960	1898	1027
17	1981	1903	1049
18	1994	1886	1054
19	1989	1896	1054
20	1990	1882	—

Reaction of the complexes 1–4 with HBF_4_·Et_2_O in dichloromethane at −40 °C afforded the complexes bearing η^2^-coordinated propene ligand [{η^5^:κ*S*-C_5_H_4_(CH_2_)_2_SR}M(CO)_2_(η^2^-CH_2_CHCH_3_)][BF_4_] (Scheme 2).

The absence of coordinating solvents enabled isolation of molybdenum complexes 5 and 6 at low temperature. They were stored for days at −40 °C without signs of decomposition. We note that they were characterized by ^1^H NMR spectroscopy only owing to their thermal instability.

The ^1^H NMR spectra of complexes 5 and 6, measured at room temperature, contained two sets of signals attributed to two isomeric species of [{η^5^:κ*S*-C_5_H_4_(CH_2_)_2_SR}Mo(CO)_2_(η^2^-C_2_H_3_Me)][BF_4_] with different propene ligand orientations (Scheme 2). The origin of these two isomers is assumed to be correlated to whether the allyl ligand being protonated in 1 and 2 is in the *exo*- or *endo*-confirmation. Relative abundances of a and b isomers were determined from integration of well resolved doublets attributed to propene methyl groups (*δ* = 1.8–2.3 ppm). The resonance at higher field was assigned to a isomer due to the effect of proximity of carbonyl ligand.

In both cases (5 and 6), isomer b is formed as a major protonation product since the spectra measured immediately upon dissolution contain isomers 5a/5b and 6a/6b in molar ratio 4 : 5 and 3 : 5, respectively. The lower thermodynamic stability of the b isomers become evident from repeated measurements. After two hours at room temperature, the composition of the mixtures has changed considerably. The isomers 5a/5b and 6a/6b were observed in molar ratios 6 : 1 and 5 : 1, respectively. We note that the rearrangement of a to b is accompanied with slow decomposition of the propene complexes. After prolonged storage at room temperature, full decomposition was evidenced by disappearance of signals attributed to a and b and detection of free propene giving characteristic signal at 1.70 ppm (dt, ^3^*J*(^1^H,^1^H) = 6.5 Hz, ^4^*J*(^1^H,^1^H) = 1.5 Hz).

Conversion of a to b, was verified by NMR measurement with internal standard. Such experiments have shown the increase of a isomer concentration up to 130% and 160% of original concentration for 5a and 6a, respectively.

The tungsten complexes 7 and 8 are thermally stable. They were isolated at room temperature and can be long-term stored without signs of decomposition. The thermodynamic stability of tungsten-propene bond was further evidenced by their inertness toward coordinating solvents (*e.g.*, MeCN) and aromatic amines (*e.g.*, pyridine) including strong *N*,*N*-chelators (*e.g.*, 1,10-phenanthroline).

The complexes 7 and 8 were characterized by mass spectrometry, ^1^H NMR, ^13^C NMR and IR spectroscopy. In both cases, only one set of signals was observed in ^1^H NMR spectra attributed to single isomer (presumably isomer a). The coordinated propene ligand gives three signals assigned to methyl group (doublet at ∼2.1 ppm), methylidene group (7: 3.05 ppm; 8: 2.64 ppm) and alkene CH group (7: 3.52 ppm; 8: 3.22 ppm), with the use ^1^H–^1^H COSY technique. The ^1^H NMR spectra also contained four signals of the cyclopentadienyl CH groups and four multiplets of ethylene chain, which implies intramolecular coordination of the thioether sulfur atom of the side chain. Low symmetry of the compounds 7 and 8 is also apparent from the ^13^C NMR spectra. For instance, each of the two carbonyl ligands gives an independent signal (7: 213.3 and 214.3 ppm; 8: 214.0 and 214.7 ppm). Detailed assignment of ^13^C NMR spectra was done with use of ^1^H–^13^C HSQC technique.

The successful protonation of the allyl ligand is also evidenced in the IR spectra. The carbonyl stretching bands are shifted by ∼90 cm^−1^ to higher wavenumbers compared to the precursor complexes 3 and 4 (Table 1).

**Fig. 1:**
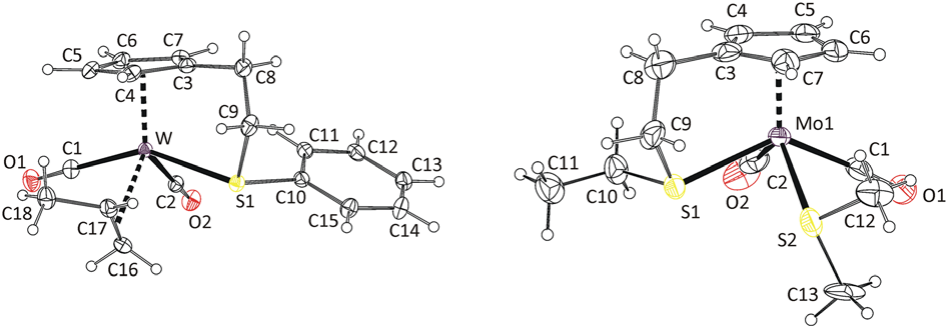
X-ray structure of 8a (left) and 9-*cis* (right). Thermal ellipsoids set to 30% probability. Only one of the three crystallographically independent cations of 9-*cis* is shown for clarity.

Formation of the isomer 8a was confirmed by X-ray diffraction analysis on a single crystal (Fig. 1). The coordination sphere of the tungsten atom adopts a distorted square pyramidal geometry. The apical position of the pyramid is occupied by the η^5^-coordinated cyclopentadienyl ligand. The square base contains the two carbonyl ligands in *cis*-configuration, the intramolecularly coordinated sulfur atom of the thioether moiety and the C=C double bond of the propene ligand. Methyl group of propene ligand appears at *cis*-position with respect to neighboring carbonyl ligand C1O1 (isomer a in Scheme 2). Selected bond lengths and angles are presented in Table 2.

**Table 2:** Selected bond lengths (Å) and angles (°) of molybdenum and tungsten complexes

	8a	9-*cis*^a^	9-*cis*^a^	9-*cis*^a^	11-*cis*	12-*cis*	15-*trans*	16-*cis*	18	19a
M–Cg(C_5_)^b^	1.9877(15)	1.974(4)	1.984(4)	1.984(5)	1.9797(15)	1.9820(9)	2.0023(18)	1.9880(11)	1.9820(11)	1.9871(8)
M–C1	2.027(3)	1.99(1)	2.00(1)	2.01(1)	2.006(4)	2.000(2)	2.017(4)	1.975(2)	1.998(3)	1.984(2)
M–C2	1.983(3)	1.94(1)	1.95(1)	1.96(1)	1.964(3)	1.963(2)	1.959(4)	1.965(2)	1.951(2)	1.981(2)
M–L1^c^	2.5362(8)	2.509(2)	2.519(2)	2.516(2)	2.516(1)	2.5232(5)	2.505(1)	2.5416(5)	2.5120(7)	2.166(1)
M–L2^d^	2.210(2)	2.546(3)	2.530(2)	2.489(3)	2.5147(9)	2.5314(5)	2.490(1)	2.5395(6)	2.163(2)	2.168(1)
C1–M–Cg(C_5_)	105.53(11)	117.9(3)	110.9(3)	113.5(4)	117.67(11)	113.30(6)	134.23(13)	114.69(8)	118.54(10)	116.30(5)
C2–M–Cg(C_5_)	123.04(10)	122.4(4)	123.7(3)	126.6(3)	122.82(11)	125.97(6)	123.48(11)	115.15(7)	121.28(7)	117.91(5)
L1–M–Cg(C_5_)^b^^,^^c^	104.41(5)	107.75(16)	106.60(14)	106.07(16)	107.42(5)	107.93(3)	105.68(7)	120.36(4)	107.20(4)	113.91(4)
L2–M–Cg(C_5_)^b^^,^^d^	133.96(8)	118.14(17)	124.13(14)	123.37(16)	118.92(5)	120.98(3)	106.40(7)	121.60(4)	120.21(6)	116.86(4)
C1–M–L1^c^	149.8(1)	134.4(3)	142.0(3)	140.3(3)	134.7(1)	138.43(6)	147.85(3)	124.84(7)	134.17(9)	129.78(6)
C2–M–L2^d^	102.9(1)	119.4(3)	112.2(3)	109.8(3)	118.1(1)	113.03(6)	102.3(2)	123.23(6)	118.41(8)	125.23(6)
C1–M–C2	79.1(1)	74.3(5)	78.5(4)	77.7(4)	75.4(1)	76.74(8)	102.3(2)	75.66(9)	75.2(1)	74.85(7)
L1–M–L2^c^^,^^d^	77.53(7)	76.38(9)	75.53(8)	75.09(9)	74.61(3)	75.17(2)	147.85(3)	74.98(2)	75.15(5)	77.47(5)

a Three crystallographically independent molecules in the unit cell.

b Cg(C_5_) = center of the cyclopentadienyl ring.

c L1 = sulfur atom of the pendant arm (8a, 9-*cis*, 11-*cis*, 12-*cis* and 18), sulfur atom of the monodentate thioether (15-*trans*, 16-*cis*) or the nitrogen atom of the acetonitrile (19a).

d L2 = center of the C=C double bond of propene (8a), sulfur atom of the monodentate thioether (9-*cis*, 11-*cis*, 12-*cis*, 15-*trans*, 16-*cis*) or the nitrogen atom of the acetonitrile (18, 19a).

It is well established that the C=C double bond of the alkene ligand elongates upon coordination and that the substituents on the C=C carbon atoms bend away from the metal atom. This appears because of the sharing of electron density between the π-orbitals of the alkene and the orbitals of the metal center.^21^ The dihedral angle defined by the metal atom, the C=C carbon atoms and carbon atom of the propene methyl group (*α*) can be used for quantifying distortion of the propene ligand. Its deviation from 90° express degree of its bending (*α*′ = *α* − 90). Elongation of the C=C double bond in η^2^-propene complexes usually vary between 0.03–0.13 Å (ref. 24–27) compared to value determined for free propene molecule (1.341(2) Å) by the gas electron diffraction.^28^ In the case of compound 8a, the C=C bond elongates by 0.057 Å, which is comparable to data previously reported for indenyl tungsten(ii) complex [{η^5^:κ*N*-1-(C_9_H_6_N)C_9_H_6_}(η^2^-C_2_H_3_Me)W(CO)_2_][BF_4_]^21^ (0.068 Å) and calixarene tungsten(iv) complex [{*p*-Bu^t^-calix[4]-(O)_4_}W(η^2^-C_2_H_3_Me)], (0.058 Å).^25^

Complexes of electron rich metals such as Pt(ii)^29,30^ and Cu(i)^24^ show low values of bending angle α′ not exceeding 15°. It implies a low effect of the coordination distortion on geometry of the propene ligand. The complexes of less electron rich metals, such as Ta(iii),^27^ Mo(ii),^26^ W(ii)^21,31^ and W(iv),^25^ show higher α′ values. They vary between 21° and 30°, which documents a stronger effect of the coordination.

In our case (8a), the bending angle (21.9(3)°) fits into this range being close to value recently reported to structurally related indenyl tungsten(ii) compound [{η^5^:κ*N*-1-(C_9_H_6_N)C_9_H_6_}(η^2^-C_2_H_3_Me)W(CO)_2_][BF_4_]^21^ (24.5(9)°).

**Scheme 3:**
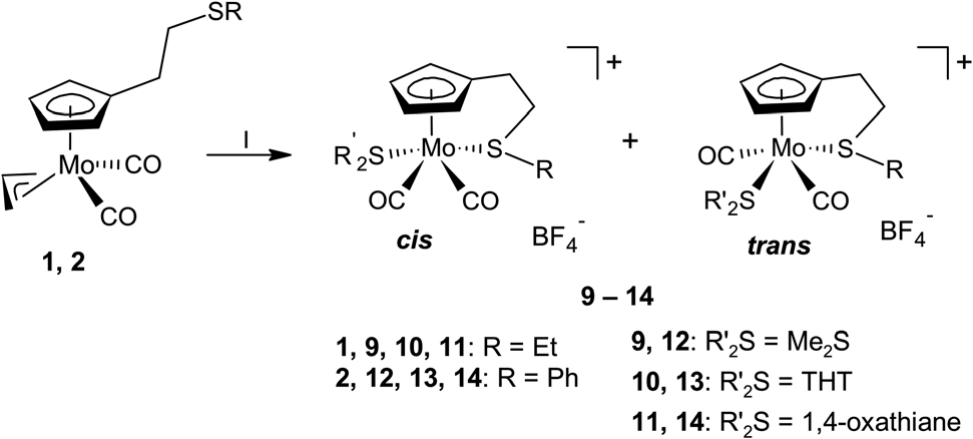
Protonation of 1 and 2 in the presence of thioethers: (I) HBF_4_·Et_2_O, CH_2_Cl_2_, 1.1 equiv. 
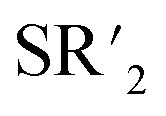
, 0 °C.

To assess the stability of molybdenum(ii)-to-alkene bond in 5 and 6, protonation of their precursors (1 and 2) was done in presence of weak coordinating thioether ligands (Scheme 3). Infrared spectra of the isolated products 9–14 revealed shift of the carbonyl stretching bands by ∼40 cm^−1^ to higher wavenumbers compared to the precursors 1 and 2 (Table 1), which documents lower covalency of the Mo–CO bond in the cationic products due to reduced π-backbonding of the carbonyl ligands.

^1^H NMR spectra of the compounds 9–14 contain four sharp signals at 5.0–6.5 ppm assigned to hydrogen atoms of the cyclopentadienyl ring. It indicates that the sulfur atom of the thioether side chain is intramolecularly coordinated. Additionally, the spectra of compounds 9, 10 and 13 contained a second set of four signals in this region implying a presence of two isomeric species. They appear in molar ratios 5 : 1, 13 : 1 and 5 : 2 in solutions of 9, 10 and 13, respectively. The presence of two isomers is also evidenced in the ^13^C NMR spectra of 9, 10 and 13. They contain two sets of signals for the carbon atoms of the carbonyl ligands. The ^1^H and ^13^C NMR spectra indicate low molecular symmetry of both isomers due to coordination of thioether side arm. The existence of two discrete isomeric species is also confirmed by the presence of two separate sets of signals for the ethylene spacers, observed in the ^1^H NMR spectrum of 13, which were assigned using the ^1^H–^1^H COSY technique. We note that signals of the ethylene spacer were not unambiguously assigned in the ^1^H NMR spectra of 9 and 10 owing to the presence of other aliphatic functions.

Molecular structure of the compound 9-*cis* was revealed by X-ray diffraction analysis on a single crystal (Fig. 1). The compound has the expected square-pyramidal structure with carbonyl ligands in expected *cis*-configuration. Such evidence together with similarities observed in NMR spectra led us to attribute the main product in spectra of 9–14 to the same isomer (see, *cis* in Scheme 3). The minor species, in the spectra of 9, 10 and 13, is ascribed to the isomer with the carbonyl ligands in the *trans*-configuration. Although this configuration is less common for compounds of general formula [Cp′M(CO)_2_L_2_], it was previously reported for derivatives bearing thioether ligands without intramolecular coordination.^32^

**Fig. 2:**
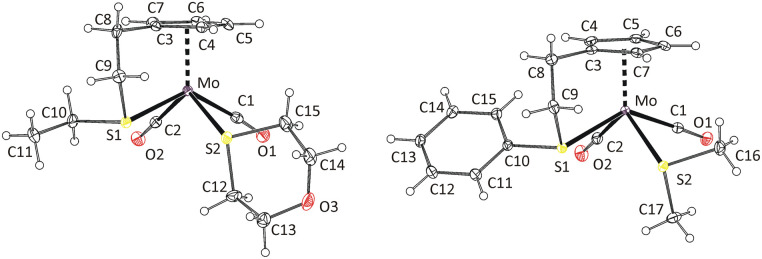
X-ray structure of 11-*cis* (left) and 12-*cis* (right). Thermal ellipsoids set to 30% probability.

The formation of *cis*-isomers was evidenced by X-ray analysis also for compounds 11-*cis* and 12-*cis* (Fig. 2). They are isostructural with compound 9-*cis* mentioned afore. Presence of simple thioether ligand in these structures enables to quantify effects of geometric constrain, caused by intramolecularly coordinated side arm. As evident from data given in Table 2, the intramolecular coordination causes only minor shortening of the Mo–S bond but the reduction of the bond angle Cg(C_5_)–Mo–S is substantial (from ∼120 to ∼107°).

**Scheme 4:**
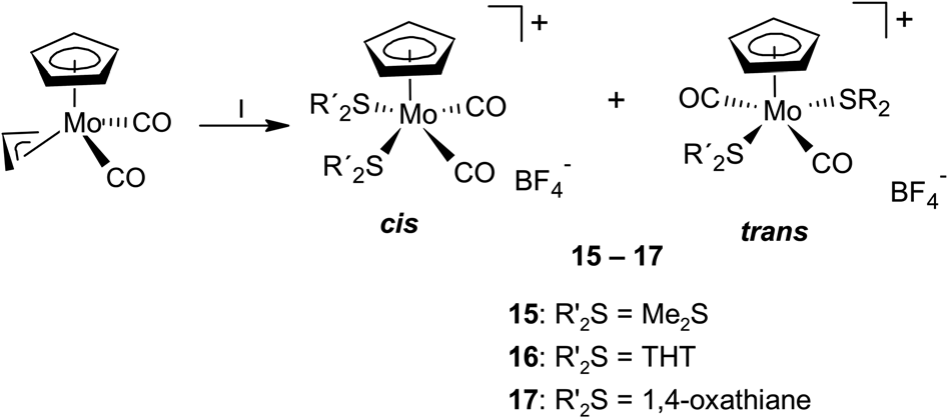
Synthesis of the thioether complexes 15–17: (I) HBF_4_·Et_2_O, CH_2_Cl_2_, 2.2 equiv. 
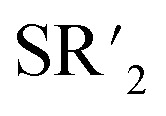
, 0 °C.

**Fig. 3:**
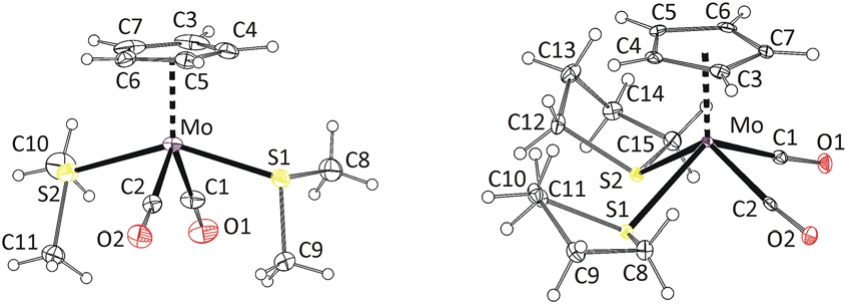
X-ray structure of 15-*trans* (left) and 16-*cis* (right). Thermal ellipsoids set to 30% probability.

Observation of the rather unusual *trans*-isomers in solutions of 10, 12 and 13 let us to investigate protonation of parent allyl complex [(η^5^-C_5_H_5_)Mo(CO)_2_(η^3^-C_3_H_5_)] in the presence of simple thioethers (Scheme 4). Infrared spectra of reaction products 15–17 show carbonyl stretching bands at similar wavenumbers as the compounds with intramolecular coordination (Table 1). ^1^H NMR spectra contain one singlet at 5.7 ppm assigned to cyclopentadienyl hydrogens, and one set of signals of given coordinated thioether. The integral intensities of the signals prove that two thioether ligands are coordinated. ^13^C NMR spectra of 15–17 contain a sole signal in the region of carbonyl ligands at ∼244 ppm, which is in line with expected *C*_s_ molecular symmetry. Unfortunately, our NMR experiments are not conclusive about configuration of carbonyl ligands. They imply formation of a single isomer or a fast equilibrium between the *cis*- and *trans*-isomers. The later interpretation in more convenient, as representatives of both isomers (15-*trans* and 16-*cis*) were evidenced by X-ray crystallography (Fig. 3).

**Fig. 4:**
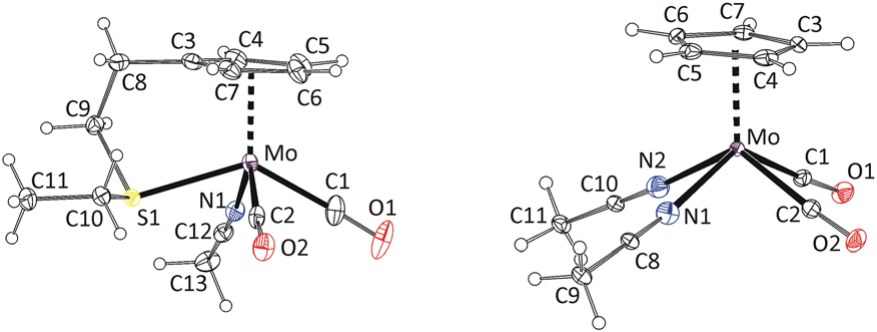
X-ray structure of 18 (left) and 20a (right). Thermal ellipsoids set to 30% probability.

Complex cations in 15-*trans* and 16-*cis* adopt expected distorted square pyramidal structures with cyclopentadienyl ligand in the apical position. Two carbonyl ligands and sulfur atoms of thioethers occupy the basal plane. The *trans-*configuration of the carbonyl ligands in 15-*trans* causes a greater distortion of the square pyramidal geometry than is observable for the cationic complexes in *cis*-configuration. This is evident from the values of the C1–Mo–C2 (102.3(2)°) and S1–Mo–S2 (147.85(3)°) angles. They differ by ∼45°, which contrasts with the structures of the other two complexes with unsubstituted cyclopentadienyl ligands (Fig. 3 and 4). The difference between C1–M–L1 and C2–M–L2 is only 1.61° and 4.55° in structures 16-*cis* and 19a, respectively (Table 2). Despite this difference in bond angles, there is only a negligible difference in corresponding Mo–S and Mo–C bond lengths in 15-*trans* and 16-*cis*.

The protonation of the allyl molybdenum complex 1 was further studied in the presence of acetonitrile. Such ligand is usually coordinated to molybdenum(ii) stronger than thioethers^21^ but the coordination is weak enough to undergo of ligand exchange reactions. Hence, acetonitrile complexes are known as stable precursors for the assembly variety of structural motifs including bis(cyclopentadienyl) compounds^33^ or complexes bearing *N*,*N*-bidentate ligands.^34^

**Scheme 5:**
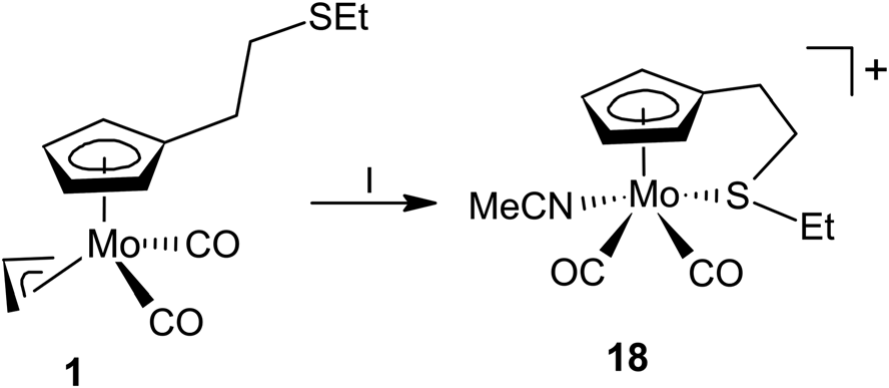
Synthesis of acetonitrile complex 18: (I) HBF_4_·Et_2_O, acetonitrile, 0 °C.

Compounds 1 reacts with HBF_4_·Et_2_O in acetonitrile to give cationic complex 18 (Scheme 5). The carbonyl stretching bands in the IR spectrum of 18 are shifted by about 40 cm^−1^ to higher energy compared to the precursor complex 1, which resembles behavior of the thioether complexes (Table 1).

The ^1^H NMR spectrum of compound 18 contains four sharp signals in the region 5.0–6.5 ppm, those were assigned to the hydrogen atoms of the cyclopentadienyl ring. It further contains four multiplets (2.62, 2.96, 3.68 and 4.08 ppm) attributed to the hydrogen atoms of the methylene spacers and signals for the ethyl group (1.39 and 2.88 ppm). Such pattern is very similar to analogues 9–11 mentioned afore, which implies appearance of the same structural motif. Signal of acetonitrile ligand appears at 2.54 ppm. Its integral intensity proves that only one molecule of the ligand is coordinated.

The structure of 18 was confirmed by X-ray diffraction analysis (Fig. 4). The coordination sphere adopts a distorted square pyramidal geometry resembling other cationic complexes under the study. The carbonyl ligands are in expected *cis*-configuration. The geometrical constrain induced by the intramolecular coordination of thioether function Cg(C_5_)–Mo–S angle (107.20(4)°) is comparable to that in crystal structures of 8, 9-*cis*, 11-*cis* and 12-*cis* (Table 2).

**Scheme 6:**
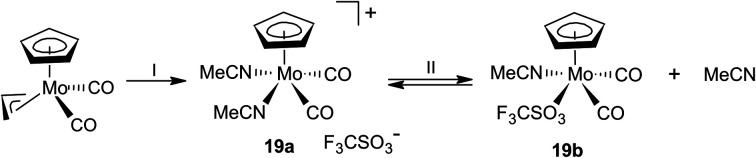
Synthesis of 19a and its transformation to 19b: (I) CF_3_SO_3_H, MeCN, 0 °C (II) CD_2_Cl_2_.

As protonation of [(η^5^-C_5_H_5_)Mo(CO)_2_(η^3^-C_3_H_5_)] by HBF_4_·Et_2_O in presence of acetonitrile is well described in literature,^35,36^ we decided to study the protonation with another strong acid CF_3_SO_3_H. The reaction was done in acetonitrile at 0 °C. ^1^H NMR spectrum in CD_2_Cl_2_ revealed a formation of two molybdenum complexes 19a and 19b in solution (Scheme 6). They give two sets of signals assigned to cyclopentadienyl (5.74 ppm for b and 5.75 ppm for a) and the coordinated acetonitrile ligands (2.50 ppm for b and 2.54 ppm for a). The spectrum further contains a singlet at 1.97 ppm which originates from free acetonitrile. It implies that recrystallization from acetonitrile produces [(η^5^-C_5_H_5_)Mo(CO)_2_(MeCN)_2_](CF_3_SO_3_) (19a) in a pure form and dissolution in CD_2_Cl_2_ leads to partial exchange of acetonitrile ligand with triflate. Such interpretation well correlates with formation equimolar amounts of 19b and free acetonitrile, evidenced by the ^1^H NMR technique.

Single crystal of 19a, prepared by a slow diffusion of diethyl ether into an acetonitrile solution, enabled to verify its solid-state structure (Fig. 4). We note that geometric parameters describing coordination sphere of molybdenum (Table 2) are in line with those reported previously for [(η^5^-C_5_H_5_)Mo(CO)_2_(NCMe)_2_][BF_4_].^35^

## Conclusions

This study describes protonation of allyl ligand in molybdenum and tungsten complexes [(η^5^-Cp′)(η^3^-allyl)M(CO)_2_] (M = Mo, W) bearing cyclopentadienyl ligand decorated with a thioether function in the side chain. After allyl ligand protonation, the thioether moiety coordinates intramolecularly to the central metal and stabilizes the η^2^-bond of appeared propene ligand.

Molybdenum complexes [{η^5^:κ*S*-C_5_H_4_(CH_2_)_2_SR}Mo(CO)_2_(η^2^-C_2_H_3_Me)][BF_4_] (R = Et, Ph), formed by this pathway, are isolable at low temperature while tungsten analogues are long-term stable at room temperature. Unusually high thermodynamic stability of tungsten complexes is documented by inertness toward coordinating solvents (*e.g.*, acetonitrile) and strong chelators (*e.g.*, 1,10-phenanthroline). In the case of less stable molybdenum compounds, η^2^-propene ligand can be easily exchanged even by labile ligands (*e.g.*, thioethers). The products of ligand exchange [{η^5^:κ*S*-C_5_H_4_(CH_2_)_2_SR}Mo(CO)_2_
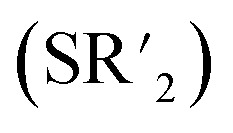
][BF_4_] form both *cis-* and *trans*-isomers in solution, though structure of *cis*-isomers were resolved by X-ray crystallography. Rather unusual *trans*-isomer was structurally characterized in the case of analoque without intramolecular coordination [(η^5^-C_5_H_5_)Mo(CO)_2_(SMe_2_)_2_][BF_4_] formed by protonation of cyclopentadienyl complex [(η^5^-C_5_H_5_)Mo(CO)_2_(η^3^-C_3_H_5_)] in the presence of dimethyl sulfide.

## Experimental

### Materials

Synthesis of the organometallic compounds was done under an argon atmosphere using conventional Schlenk-line techniques. The products were stored under argon atmosphere at −20 °C. The solvents were dried using standard methods.^37^ The reagents were purchased from comercial sources (Sigma-Aldrich, Acros Organics and Penta) or prepared according to literature procedures: [(η^5^-C_5_H_5_)Mo(CO)_2_(η^3^-C_3_H_5_)];^38^ [(η^3^-C_3_H_5_)(MeCN)_2_Mo(CO)_2_Cl];^39^ [(η^3^-C_3_H_5_)(EtCN)_2_W(CO)_2_Cl].^40^

### Methods

^1^H, ^13^C{^1^H}, ^13^C-APT, ^1^H–^1^H COSY and ^1^H–^13^C HSQC NMR spectra were measured on the Bruker Avance 500 MHz and Bruker Avance 400 MHz spectrometers. The spectra were calibrated to the residual signal of the solvent relative to Me_4_Si. Poly(dimethylsiloxane) was used as internal standard in stability studies. Infrared spectra were recorded on a Nicolet iS50 FTIR spectrometer using a diamond Smart Orbit ATR in the region 4000–400 cm^−1^. Mass spectra were collected on a quadruple mass spectrometer LCMS 2010 (Shimadzu, Japan). The samples were dissolved in acetone and injected into the mass spectrometer with an infusion mode at a constant flow rate of 10 μL min^−1^. Electrospray ionization-mass spectrometry (ESI-MS) was used for the identification of the analyzed samples.

### X-ray crystallography

Data for 9-*cis*, 11-*cis*, 12-*cis*, 16-*cis* and 19a was collected on the Rigaku XtaLAB Synergy S diffractometer equipped with micro-focus CuKα/MoKα radiation and a Hybrid Pixel Array Detector (HyPix-6000HE). An Oxford Cryosystems (Cryostream 800) cooling device was used for data collection and the crystals were kept at 100 K during data collection. CrysAlisPro software^41^ was used for data collection, cell refinement and data reduction. Data were corrected for absorption effects using empirical absorption correction (spherical harmonics), implemented in SCALE3 ABSPACK scaling algorithm and numerical absorption correction based on gaussian integration over a multifaceted crystal model. Using Olex2,^42^ the structures were solved with the SHELXT^43^ and SHELXS^44^ structure solution program using intrinsic phasing (9-*cis*, 12-*cis*, 16-*cis* and 19a) and direct methods (11-*cis*) and refined with the SHELXL^45^ refinement package using least squares minimization. Hydrogen atoms of all molecules were placed in calculated positions. The diffraction experiments for 8a, 15-*trans* and 18 were performed on Bruker D8 VENTURE Kappa Duo PHOTONIII by IμS micro-focus sealed tube CuKα (*λ* = 1.54178) radiation at low temperature. The structures were solved by direct methods (SHELXS)^42^ and refined by full matrix least squares based on *F*^2^ (SHELXL2018).^44^ The hydrogen atoms on carbon were fixed into idealized positions (riding model) and assigned atomic displacement parameters either *U*_iso_(H) = 1.2 *U*_eq_ (pivot atom) or *U*_iso_(H) = 1.5 *U*_eq_ (pivot atom) for methyl moiety.

### Synthesis of C_5_H_5_(CH_2_)_2_SEt

The substituted cyclopentadiene was prepare using modification of the previously described procedures.^46,47^

Freshly monomerized cyclopentadiene (5.2 mL, 62 mmol) was added dropwise to a suspension of sodium sand (1.6 g, 70 mmol) in 30 mL of THF. The mixture was stirred at room temperature for 16 h and then it was filtered from excess of sodium using a Schlenk frit. The sodium cyclopentadienide solution was precooled to −40 °C, treated with Cl(CH_2_)_2_SEt (7.0 g, 56 mmol) dropwise and the reaction mixture was stirred at room temperature for 16 h. The reaction was quenched with a ice/water mixture and the crude product was extracted with diethyl ether (2 × 10 mL). The volatiles were removed on a rotavapor and the product purified by vacuum distillation (bp = 110 °C at 67 Pa). Yield: 3.9 g (19 mmol, 34%). Yellow liquid. ^1^H NMR [400 MHz, CDCl_3_, 1 : 1 mixture of 1- and 2-isomers (a : b)]: *δ* = 1.27 (t, ^3^*J*(^1^H,^1^H) = 7.4 Hz, 3H, SCH_2_C*H*_3_); 2.57 (q, ^3^*J*(^1^H,^1^H) = 7.4 Hz, 2H, SC*H*_2_CH_3_); 2.63–2.76 (m, 4H of a and 4H b, C_5_H_5_C*H*_2_C*H*_2_S); 2.93 (q, ^3^*J*(^1^H,^1^H) = 1.4 Hz, 2H, H^5,5^ of a, C_5_H_5_); 2.96–2.98 (m, 2H, H^5,5^ of b, C_5_H_5_); 6.06–6.09 (m, H^4^ of a, C_5_H_5_); 6.22 (s, H^4^ of b, C_5_H_5_); 6.28 (dq, ^3^*J*(^1^H,^1^H) = 5.4 Hz, ^4^*J*(^1^H,^1^H) = 1.5 Hz, H^3^ of b, C_5_H_5_); 6.41–6.47 (m, 3H, H^3,2^ of a, H^1^ of b, C_5_H_5_).

### Synthesis of C_5_H_5_(CH_2_)_2_SPh

The steps of synthesis followed the procedure for C_5_H_5_(CH_2_)_2_SEt, but with 4.88 mL (58.0 mmol) of freshly monomerized cyclopentadiene, 1.47 g (63.9 mmol) of elemental sodium and 9.56 g (55.4 mmol) of Cl(CH_2_)_2_SPh. The desired product came over as the second fraction (100 °C, 13 mm Hg). Yield: 2.59 g (12.8 mmol, 23%). Yellow liquid. ^1^H NMR [400 MHz, CDCl_3_, 1 : 1 mixture of 1- and 2-isomers (a : b)] 2.80–2.89 (m, 2H of a and 2H of b, C_5_H_5_C*H*_2_CH_2_S); 3.03 (s, 2H, H^5,5^ of a, C_5_H_5_); 3.09 (s, 2H, H^5,5^ of b, C_5_H_5_); 3.20–3.28 (m, 2H of a and 2H of b, C_5_H_5_CH_2_C*H*_2_S); 6.22 (s, 1H, H^4^ of a, C_5_H_5_); 6.37 (s, 1H, H^4^ of b, C_5_H_5_); 6.41 (d, ^3^*J*(^1^H,^1^H) = 5.3 Hz, 1H, H^3^ of b, C_5_H_5_); 6.57 (s, 3H, H^2,3^ of a, H^1^ of b, C_5_H_5_); 7.29 (t, ^3^*J*(^1^H,^1^H) = 7.2 Hz, 2H of a and 2H of b, H^2,6^ C_6_H_5_); 7.40 (t, ^3^*J*(^1^H,^1^H) = 7.5 Hz, 2H of a and 2H of b, H^3,5^ C_6_H_5_); 7.47 (d, ^3^*J*(^1^H,^1^H) = 7.5 Hz, 1H of a and 1H of b, H^4^ C_6_H_5_).

### Synthesis of [{η^5^-C_5_H_4_(CH_2_)_2_SEt}Mo(CO)_2_(η^3^-C_3_H_5_)] (1)

C_5_H_5_(CH_2_)_2_SEt (469 mg, 3.04 mmol) was dissolved in 15 mL of THF, cooled to −60 °C and treated dropwise with a solution of butyllithium (1.6 M in hexanes, 1.9 mL, 3.04 mmol). The reaction mixture was stirred for 1 h and then added dropwise to a solution of [(η^3^-C_3_H_5_)(MeCN)_2_Mo(CO)_2_Cl] (922 mg, 2.97 mmol) in 10 mL of THF. The reaction mixture was stirred 16 h at room temperature. The volatiles were vacuum evaporated, the crude product extracted with hexane (3 × 25 mL, 60 °C). The volume of the extract was halved by vacuum evaporation and the product was precipitated as a yellow powder by cooling the mixture to −80 °C. The leftover solvent was decanted off and the product was dried in vacuum. Yield: 766 mg (2.21 mmol, 75%). Yellow powder. Anal. calc. for C_14_H_18_SO_2_Mo: C: 48.56; H: 5.24; S: 9.26. Found: C: 48.44; H: 5.10; S: 9.05. ^1^H NMR [500 MHz, CD_2_Cl_2_, 7 : 2 mixture of isomers *exo* : *endo*]: *δ* = 0.91 (d, ^3^*J*(^1^H,^1^H) = 10,8 Hz, 2H, H^anti^ of *exo*, C_3_H_5_); 1.22 (t, ^3^*J*(^1^H,^1^H) = 7.3 Hz, 3H, SCH_2_CH_3_); 1.65 (d, ^3^*J*(^1^H,^1^H) = 10.5 Hz, 2H, H^anti^ of *endo*, C_3_H_5_); 2.47 (t, ^3^*J*(^1^H,^1^H) = 7.4 Hz, 2H, C_5_H_4_CH_2_CH_2_S); 2.52 (q, ^3^*J*(^1^H,^1^H) = 7.3 Hz, 2H, SCH_2_CH_3_); 2.64 (t, ^3^*J*(^1^H,^1^H) = 7.4 Hz, 2H, C_5_H_4_CH_2_CH_2_S); 2.74 (d, ^3^*J*(^1^H,^1^H) = 7.0 Hz, 2H, H^syn^ of *exo*, C_3_H_5_); 2.78–2.83 (m, 2H, H^syn^ of *endo*, C_3_H_5_); 3.59–3.69 (m, 1H, H^meso^ of *endo*, C_3_H_5_); 3.92 (tt, ^3^*J*(^1^H,^1^H) = 10.8 Hz, ^4^*J*(^1^H,^1^H) = 7.0 Hz, 1H, H^meso^ of *exo*, C_3_H_5_); 5.17 (s, 2H, C_5_H_4_); 5.22 (s, 2H, C_5_H_4_). IR (ATR, cm^−1^): 1935 *vs.* [*ν*_a_(CO)], 1850 *vs.* [*ν*_s_(CO)].

### Synthesis of [(η^5^-C_5_H_4_(CH_2_)_2_SPh)Mo(CO)_2_(η^3^-C_3_H_5_)] (2)

The steps of the synthesis followed the procedure for 1, but with C_5_H_5_(CH_2_)_2_SPh (616 mg, 3.05 mmol), butyllithium (1.6 M in hexanes, 1.99 mL, 3.18 mmol) and [(η^3^-C_3_H_5_)(MeCN)_2_Mo(CO)_2_Cl] (925 mg, 2.98 mmol). Yield: 805 mg (2.10 mmol, 68%). Pale yellow powder. Anal. calc. for C_18_H_18_SO_2_Mo: C: 54.82; H: 4.60; S: 8.13. Found: C, 54.63; H, 4.48; S: 8.03. ^1^H NMR [500 MHz, CD_2_Cl_2_; 7 : 2 mixture of isomers *exo* : *endo*]: *δ* = 0.91 (d, ^3^*J*(^1^H,^1^H) = 10.8 Hz, 2H, H^anti^ of *exo*, C_3_H_5_); 1.63 (d, ^3^*J*(^1^H,^1^H) = 10.4 Hz, 2H, H^anti^ of *endo*, C_3_H_5_); 2.54 (t, ^3^*J*(^1^H,^1^H) = 7.5 Hz, 2H, C_5_H_5_CH_2_CH_2_SPh); 2.73 (d, ^3^*J*(^1^H,^1^H) = 6.9 Hz, 2H, H^syn^ of *exo*, C_3_H_5_); 2.80 (d, ^3^*J*(^1^H,^1^H) = 5.3 Hz, 2H, H^syn^ of *endo*, C_3_H_5_); 3.01 (t, ^3^*J*(^1^H,^1^H) = 7.5 Hz, 2H, C_5_H_5_CH_2_CH_2_SPh); 3.59–3.67 (m, 1H, H^meso^ of *endo*, C_3_H_5_); 3.90 (tt, ^3^*J*(^1^H,^1^H) = 10.7 Hz, ^3^*J*(^1^H,^1^H) = 7.1 Hz, 1H, H^meso^ of *exo*, C_3_H_5_); 5.18 (s, 2H, C_5_H_4_); 5.22 (s, 2H, C_5_H_4_); 7.20 (t, ^3^*J*(^1^H,^1^H) = 7.1 Hz, 1H, C_6_H_5_); 7.28–7.34 (m, 4H, C_6_H_5_). IR (ATR; cm^−1^): 1936 *vs.* [*ν*_a_(CO)], 1850 *vs.* [*ν*_s_(CO)].

### Synthesis of [{η^5^-C_5_H_4_(CH_2_)_2_SEt}W(CO)_2_(η^3^-C_3_H_5_)] (3)

The steps of synthesis followed the procedure for compound 1, but with C_5_H_5_(CH_2_)_2_SEt (936 mg, 6.07 mmol), butyllithium (1.6 M in hexanes, 3.8 mL, 6.08 mmol) and [(η^3^-C_3_H_5_)(EtCN)_2_W(CO)_2_Cl] (2.25 g, 5.65 mmol). Yield: 2.35 g (5.41 mmol, 96%). Orange liquid. Anal. calc. for C_14_H_18_SO_2_W: C:38.73; H: 4.18; S: 7.38. Found: C: 38.57; H: 3.94; S: 7.11. ^1^H NMR [400 MHz, C_6_D_6_, 5 : 2 mixture of isomers *exo* : *endo*]: *δ* = 1.03 (t, ^3^*J*(^1^H,^1^H) = 7.4 Hz, 3H, SCH_2_CH_3_); 1.08–1.15 (m, 2H, H^anti^ of *exo* and *endo*, C_3_H_5_); 2.12 (t, ^3^*J*(^1^H,^1^H) = 7.1 Hz, 2H, C_5_H_4_CH_2_CH_2_S); 2.19 (q, ^3^*J*(^1^H,^1^H) = 7.3 Hz, 2H, SCH_2_CH_3_); 2.26 (t, ^3^*J*(^1^H,^1^H) = 6.8 Hz, 2H, C_5_H_4_CH_2_CH_2_S); 2.38 (d, ^3^*J*(^1^H,^1^H) = 6.1 Hz, 2H, H^syn^ of *exo*, C_3_H_5_); 2.61–2.67 (m, 2H, H^syn^ of *endo*, C_3_H_5_); 2.84–2.95 (m, 1H^meso^ of *exo*, C_3_H_5_); 3.51–3.62 (m, 1H^meso^ of *endo*, C_3_H_5_); 4.50 (s, 2H, C_5_H_4_); 4.57 (s, 2H, C_5_H_4_). IR (ATR, cm^−1^): 1929 *vs.* [*ν*_a_(CO)], 1840 *vs.* [*ν*_s_(CO)].

### Synthesis of [{η^5^-C_5_H_4_(CH_2_)_2_SPh}W(CO)_2_(η^3^-C_3_H_5_)] (4)

The steps of synthesis followed the procedure for compound 1, but with C_5_H_5_(CH_2_)_2_SPh (297 mg, 1.47 mmol), butyllithium (1.6 M in hexanes, 1.0 mL, 1.6 mmol) and [(η^3^-C_3_H_5_)(EtCN)_2_W(CO)_2_Cl] (545 mg, 1.37 mmol). Yield: 498 mg (1.15 mmol, 82%). Orange liquid. Anal. calc. for C_18_H_18_SO_2_W: C: 44.83; H: 3.76; S: 6.65. Found: C: 44.55; H: 3.59; S: 6.37. ^1^H NMR [400 MHz, CDCl_3_, 2 : 1 mixture of isomers *exo* : *endo*]: *δ* = 1.19 (d, ^3^*J*(^1^H,^1^H) = 9.9 Hz, 2H, H^anti^ of *exo*, C_3_H_5_); 1.32–1.39 (m, 2H, H^anti^ of *endo*, C_3_H_5_); 2.66 (t, ^3^*J*(^1^H,^1^H) = 7.5 Hz, 2H, C_5_H_4_CH_2_CH_2_S); 2.72 (d, ^3^*J*(^1^H,^1^H) = 5.9 Hz, 2H, H^syn^ of *exo*, C_3_H_5_); 2.79–2.85 (m, 2H, H^syn^ of *endo*, C_3_H_5_); 3.13 (t, ^3^*J*(^1^H,^1^H) = 7.4 Hz, 2H, C_5_H_4_CH_2_CH_2_S); 3.46–3.56 (m, 1H, H^meso^ of *exo*, C_3_H_5_); 3.74–3.82 (m, 1H, H^meso^ of *endo*, C_3_H_5_); 5.25–5.38 (m, 4H, C_5_H_4_); 7.32–7.43 (m, 5H, C_6_H_5_). IR (ATR, cm^−1^): 1929 *vs.* [*ν*_a_(CO)], 1839 *vs.* [*ν*_s_(CO)].

### Synthesis of [{η^5^:κ*S*-C_5_H_4_(CH_2_)_2_SEt}Mo(CO)_2_(η^2^-C_2_H_3_Me)][BF_4_] (5)

Compound 1 (252 mg, 0.73 mmol) was dissolved in 10 mL of dichloromethane, the solution was cooled to −40 °C and treated with HBF_4_·Et_2_O (98 μL, 0.73 mmol) dropwise. The solution was stirred at −40 °C for 16 h, the solvent was vacuum evaporated, and the crude product was purified by dissolution in a small amount of dichloromethane (∼0.5 mL) and subsequent precipitation by the addition of diethyl ether (10 mL), the solvents were decanted and the solid was vacuum dried. This process was repeated up to three times. Care was taken to keep the reaction mixture at or below −40 °C and to measure the ^1^H NMR spectra directly after isolation. Yield: 316 mg (0.60 mmol, 82%). Orange solid. ^1^H NMR [500 MHz, CD_2_Cl_2_, 4 : 5 mixture of isomers a : b]: *δ* = 1.27 (t, ^3^*J*(^1^H,^1^H) = 7.4 Hz, 3H of b, SCH_2_C*H*_3_); 1.31 (t, ^3^*J*(^1^H,^1^H) = 7.4 Hz, 3H of a, SCH_2_C*H*_3_); 1.88 (d, ^3^*J*(^1^H,^1^H) = 5.5 Hz, 3H of a, CH_2_CHC*H*_3_); 2.21 (d, ^3^*J*(^1^H,^1^H) = 6.0 Hz, 3H of b, CH_2_CHC*H*_3_); 4.90 (s, 1H of b, C_5_H_4_); 4.95 (s, 1H of a, C_5_H_4_); 5.35 (s, 1H of b, C_5_H_4_); 6.08 (s, 1H of a, C_5_H_4_); 6.10 (s, 1H of b, C_5_H_4_); 6.25 (s, 1H of a, C_5_H_4_); 6.35 (s, 1H of b, C_5_H_4_).

### Synthesis of [{η^5^:κ*S*-C_5_H_4_(CH_2_)_2_SPh}Mo(CO)_2_(η^2^-C_2_H_3_Me)][BF_4_] (6)

The steps of synthesis followed the procedure for compound 5, but with 2 (105 mg, 0.27 mmol), HBF_4_·Et_2_O (38 μL, 0.28 mmol). Yield: 101 mg (0.21 mmol, 79%). Orange solid. ^1^H NMR [500 MHz, CD_2_Cl_2_, 3 : 5 mixture of isomers a : b]: *δ* = 1.98 (d, ^3^*J*(^1^H,^1^H) = 5.7 Hz, 3H of a, CH_2_CHC*H*_3_); 2.24 (d, ^3^*J*(^1^H,^1^H) = 6.0 Hz, 3H of b, CH_2_CHC*H*_3_); 4.96 (s, 1H of b, C_5_H_4_); 5.10 (s, 2H of a, C_5_H_4_); 5.36 (s, 1H of b, C_5_H_4_); 5.37 (s, 1H of b, C_5_H_4_); 6.33 (s, 1H of a, C_5_H_4_); 6.35 (s, 1H of a, C_5_H_4_); 6.43 (s, 1H of b, C_5_H_4_).

### Synthesis of [{η^5^:κ*S*-C_5_H_4_(CH_2_)_2_SEt}W(CO)_2_(η^2^-C_2_H_3_Me)][BF_4_] (7)

Compound 3 (731 mg, 1.68 mmol) was dissolved in 10 mL of acetonitrile, the solution was cooled to 0 °C and treated with HBF_4_·Et_2_O (250 μL, 1.85 mmol) dropwise. The solution was stirred at room temperature for 17 h, the solvent was vacuum evaporated, and the crude product was purified by dissolution in a small amount of dichloromethane (∼0.5 mL), the solution was cooled to −40 °C and subsequently the product was precipitated by the addition of diethyl ether (10 mL), the solvents were decanted and the solid was dried *in vacuo*. This process was repeated up to three times. Yield: 537 mg (1.03 mmol, 61%). Yellow solid. Anal. calc. for C_14_H_19_SO_2_WBF_4_: C: 32.21; H: 3.67; S: 6.14. Found: C: 32.01; H: 3.43; S: 5.90. Positive-ion MS (acetone): *m*/*z* (%) = 393 (100) [M − C_3_H_6_]^+^. ^1^H NMR [500 MHz, CD_2_Cl_2_]: *δ* = 1.22 (t, ^3^*J*(^1^H,^1^H) = 7.4 Hz, 3H, SCH_2_C*H*_3_); 2.09 (d, ^3^*J*(^1^H,^1^H) = 5.2 Hz, 3H, CH_2_CHC*H*_3_); 2.64 (d, ^3^*J*(^1^H,^1^H) = 10.0 Hz, 2H, C*H*_2_CHCH_3_); 2.75–2.80 (m, 1H, SC*H*_2_CH_3_); 2.82–2.89 (m, 1H, C_5_H_4_C*H*_2_CH_2_S); 2.92–3.00 (m, 1H, SC*H*_2_CH_3_); 3,12 (ddd, ^3^*J*(^1^H,^1^H) = 14.6 Hz, ^4^*J*(^1^H,^1^H) = 5.4 Hz, ^5^*J*(^1^H,^1^H) = 2.2 Hz, 1H, C_5_H_4_C*H*_2_CH_2_S); 3.18–3.26 (m, 1H, CH_2_C*H*CH_3_); 3.77 (td, ^3^*J*(^1^H,^1^H) = 13.7 Hz, ^4^*J*(^1^H,^1^H) = 5.5 Hz, 1H, C_5_H_4_CH_2_C*H*_2_S); 3.89 (ddd, ^3^*J*(^1^H,^1^H) = 10.9 Hz, ^4^*J*(^1^H,^1^H) = 4.7 Hz, ^5^*J*(^1^H,^1^H) = 2.8 Hz, 1H, C_5_H_4_CH_2_C*H*_2_S); 5.01 (m, 1H, C_5_H_4_); 5.08 (s, 1H, C_5_H_4_); 6.24 (m, 1H, C_5_H_4_); 6.37 (s, 1H, C_5_H_4_). ^13^C NMR [126 MHz, CD_2_Cl_2_]: *δ* = 13.6 (SCH_2_CH_3_); 25.9 (CH_2_CHCH_3_); 26.5 (C_5_H_4_CH_2_CH_2_S); 34.8 (SCH_2_CH_3_); 45.0 (CH_2_CHCH_3_); 51.4 (C_5_H_4_CH_2_CH_2_S); 51.8 (CH_2_CHCH_3_); 76.1 (C_5_H_4_); 85.1 (C_5_H_4_); 88.0 (C_5_H_4_); 94.0 (C_5_H_4_); 139.8 (C_5_H_4_); 214.0 (CO); 214.7 (CO). IR (ATR, cm^−1^): 2025 *vs.* [*ν*_a_(CO)], 1946 *vs.* [*ν*_s_(CO)], 1034 *vs.* [*ν*(BF)].

### Synthesis of [{η^5^:κ*S*-C_5_H_4_(CH_2_)_2_SPh}W(CO)_2_(η^2^-C_2_H_3_Me)][BF_4_] (8)

The steps of synthesis followed the procedure for compound 7, but with 4 (135 mg, 0.28 mmol) and HBF_4_·Et_2_O (40 μL, 0.30 mmol). Yield: 102 mg (0.18 mmol, 64%). Yellow solid. Anal. calc. for C_18_H_19_SO_2_WBF_4_: C: 37.93; H: 3.36; S: 5.62. Found: C: 37.60; H: 3.14; S: 5.35. Positive-ion MS (acetone): *m*/*z* (%) = 441 (100) [M – C_3_H_6_]^+^. ^1^H NMR [500 MHz, CD_2_Cl_2_]: *δ* = 2.14 (d, ^3^*J*(^1^H,^1^H) = 4.7 Hz, 3H, CH_2_CHC*H*_3_); 2.45–2.57 (m, 1H, C_5_H_4_C*H*_2_CH_2_S); 2.66 (dd, ^2^*J*(^1^H,^1^H) = 11.8 Hz, ^3^*J*(^1^H,^1^H) = 5.3 Hz, 1H, C_5_H_4_C*H*_2_CH_2_S); 2.99–3.11 (m, 2H, C*H*_2_CHCH_3_); 3.52 (s, 1H, CH_2_C*H*CH_3_); 3.97–4.04 (m, 1H, C_5_H_4_CH_2_C*H*_2_S); 4.12–4.19 (m, 1H, C_5_H_4_CH_2_C*H*_2_S); 5.07 (s, 1H, C_5_H_4_); 5.16 (s, 1H, C_5_H_4_); 6.45 (s, 1H, C_5_H_4_); 6.53 (s, 1H, C_5_H_4_); 7.51 (5H, C_6_H_5_). ^13^C NMR [126 MHz, CD_2_Cl_2_]: *δ* = 25.9 (CH_2_CHCH_3_); 27.5 (CH_2_CHCH_3_); 45.5 (CH_2_CHCH_3_); 51.6 (C_5_H_4_CH_2_CH_2_S); 57.9 (C_5_H_4_CH_2_CH_2_S); 76.9 (C_5_H_4_); 84.4 (C_5_H_4_); 86.9 (C_5_H_4_); 95.1 (C_5_H_4_); 129.4 (C_6_H_5_); 130.7 (C_6_H_5_); 132.1 (C_6_H_5_); 139.0 (C_5_H_4_); 213.3 (CO); 214.3 (CO). IR (ATR, cm^−1^): 2025 *vs.* [*ν*_a_(CO)], 1915 *vs.* [*ν*_s_(CO)], 1026 *vs.* [*ν*(BF)]. Single crystals of 8 suitable for X-ray diffraction analysis were prepared by overlayering of the acetonitrile solution of 8 with diethyl ether.

### Synthesis of [{η^5^:κ*S*-C_5_H_4_(CH_2_)_2_SEt}Mo(CO)_2_(SMe_2_)][BF_4_] (9)

Compound 1 (119 mg, 0.34 mmol) was dissolved in 10 mL of dichloromethane, the solution was treated with Me_2_S (50 μL, 0.68 mmol) cooled to 0 °C and treated with HBF_4_·Et_2_O (46 μL, 0.34 mmol) dropwise. The color of the solution changed from orange to red immediately upon the addition of acid. The reaction mixture was stirred at room temperature for 16 h, the solvent was vacuum evaporated, and the crude product was purified by dissolution in a small amount of dichloromethane (∼0.5 mL), the solution was cooled to −40 °C and subsequently the product was precipitated by the addition of diethyl ether (10 mL), the solvents were decanted and the solid was dried *in vacuo*. This process was repeated up to three times. Yield: 122 mg (0.27 mmol, 79%). Red solid. Anal. calc. for C_13_H_19_S_2_O_2_MoBF_4_: C: 34.38; H: 4.22; S: 14.12. Found: C: 34.20; H: 4.07; S: 13.88. Positive-ion MS (acetone): *m*/*z* (%) = 369 (100) [M]^+^. ^1^H NMR [500 MHz, CD_2_Cl_2_, mixture 5 : 1 of isomers *cis* : *trans*]: *δ* = 1.38 (t, ^3^*J*(^1^H,^1^H) = 7.3 Hz, 3H, SCH_2_C*H*_3_); 2.52 (s, 6H, (CH_3_)_2_S); 2.65–2.75 (m, 1H, C_5_H_4_C*H*_2_CH_2_S); 2.89–2.99 (m, 3H, 1H of C_5_H_4_C*H*_2_CH_2_S; 2H of SC*H*_2_CH_3_); 3.71–3.78 (m, 1H, C_5_H_4_CH_2_C*H*_2_S); 3.81–3.88 (m, 1H, C_5_H_4_CH_2_C*H*_2_S); 4.88–4.90 (m, 1H, C_5_H_4_ of *cis*); 5,03 (m, 1H, C_5_H_4_ of *trans*); 5.16–5.19 (m, 1H, C_5_H_4_ of *trans*); 5.35–5.36 (m, 1H, C_5_H_4_ of *trans*); 5.56 (s, 1H, C_5_H_4_ of *cis*); 5.64 (s,1H, C_5_H_4_ of *cis*); 6.19–6.10 (m, 1H, C_5_H_4_ of *trans*); 6.29 (s, 1H, C_5_H_4_ of *cis*). ^13^C NMR [126 MHz, CD_2_Cl_2_]: *δ* = 13.7 (SCH_2_CH_3_ of *cis*); 13.9 (SCH_2_CH_3_ of *trans*); 25.9 (SCH_2_CH_3_); 28.2 ((CH_3_)_2_S of *cis*); 28.5 ((CH_3_)_2_S of *trans*); 34.1 (C_5_H_4_CH_2_CH_2_S of *cis*); 36.3 (C_5_H_4_CH_2_CH_2_S of *trans*); 54.0 (C_5_H_4_CH_2_CH_2_S of *cis*); 66.2 (C_5_H_4_CH_2_CH_2_S of *trans*); 82.8 (C_5_H_4_ of *cis*); 87.5 (C_5_H_4_ of *cis*); 89.2 (C_5_H_4_ of *cis*); 89.8 (C_5_H_4_ of *trans*); 92.7 (C_5_H_4_ of *trans*); 93.9 (C_5_H_4_ of *trans*); 95.4 (C_5_H_4_ of *trans*); 100.4 (C_5_H_4_ of *cis*); 130.9 (C_5_H_4_ of *trans*); 142.3 (C_5_H_4_ of *cis*); 230.6 (CO of *trans*); 235.8 (CO of *trans*); 241.8 (CO of *cis*); 244.2 (CO of *cis*). IR (ATR, cm^−1^): 1975 *vs.* [*ν*_a_(CO)], 1883 *vs.* [*ν*_s_(CO)], 1019 *vs.* [*ν*(BF)]. Single crystals of 9-*cis* suitable for X-ray diffraction analysis were prepared by the overlayering of dichloromethane solution of 9 with diethyl ether.

### Synthesis of [{η^5^:κ*S*-C_5_H_4_(CH_2_)_2_SEt}Mo(CO)_2_{S(CH_2_)_4_}][BF_4_] (10)

The steps of synthesis followed the procedure for compound 9, but with 1 (176 mg, 0.51 mmol), tetrahydrothiophene (67 μL, 0.76 mmol) and HBF_4_·Et_2_O (70 μL, 0.52 mmol). Yield: 186 mg (0.39 mmol, 76%). Red solid. Anal. calc. for C_15_H_21_S_2_O_2_MoBF_4_: C: 37.52; H: 4.41; S: 13.35. Found: C: 37.27; H: 4.19; S: 13.09. ^1^H NMR [500 MHz, CD_2_Cl_2_, mixture 13 : 1 of isomers *cis* : *trans*]: *δ* = 1.37 (t, ^3^*J*(^1^H,^1^H) = 7.3 Hz, 3H, SCH_2_C*H*_3_); 2.10–2.19 (m, 4H, H^3,3,4,4^, C_4_H_8_S); 2.70–2.78 (m, 1H, C_5_H_4_C*H*_2_CH_2_S); 2.87–3.02 (m, 3H, 1H of C_5_H_4_C*H*_2_CH_2_S, 2H of SC*H*_2_CH_3_); 3.03–3.20 (m, 4H, H^2,2,5,5^, C_4_H_8_S); 3.70–3.85 (m, 2H, C_5_H_4_CH_2_C*H*_2_S); 4.87–4.89 (m, 1H, C_5_H_4_ of *cis*); 5.06 (m, 1H, C_5_H_4_ of *trans*); 5.11 (m, 1H, C_5_H_4_ of *trans*); 5.54 (s, 1H, C_5_H_4_ of *cis*); 5.72 (s, 1H, C_5_H_4_ of *cis*); 6.09 (m, 1H, C_5_H_4_ of *trans*); 6.12 (m, 1H, C_5_H_4_ of *trans*); 6.23 (s, 1H, C_5_H_4_ of *cis*). ^13^C NMR [126 MHz, CD_2_Cl_2_]: *δ* = 13.8 (SCH_2_CH_3_ of *cis*); 15.5 (SCH_2_CH_3_ of *trans*); 25.3 (SCH_2_CH_3_ of *trans*); 25.8 (SCH_2_CH_3_ of *cis*); 31.0 (C^3,4^, C_4_H_8_S of *trans*); 31.2 (C^3,4^, C_4_H_8_S of *cis*); 34.0 (C_5_H_4_CH_2_CH_2_S); 44.4 (C^2,5^, C_4_H_8_S of *trans*); 45.0 (C^2,5^, C_4_H_8_S of *cis*); 54.0 (C_5_H_4_CH_2_CH_2_S); 83.2 (C_5_H_4_ of *cis*); 87.4 (C_5_H_4_ of *cis*); 89.0 (C_5_H_4_ of *cis*); 89.9 (C_5_H_4_ of *trans*); 92.6 (C_5_H_4_ of *trans*); 93.9 (C_5_H_4_ of *trans*); 95.0 (C_5_H_4_ of *trans*); 100.6 (C_5_H_4_ of *cis*); 131.1 (C_5_H_4_ of *trans*); 142.1 (C_5_H_4_ of *cis*); 231.4 (CO of *trans*); 235.3 (CO of *trans*); 242.7 (CO of *cis*); 245.1 (CO of *cis*). IR (ATR, cm^−1^): 1974 *vs.* [*ν*_a_(CO)], 1882 *vs.* [*ν*_s_(CO)], 1048 *vs.* [*ν*(BF)].

### Synthesis of [{η^5^:κ*S*-C_5_H_4_(CH_2_)_2_SEt}Mo(CO)_2_{κ*S*-S(CH_2_)_4_O}][BF_4_] (11)

The steps of synthesis followed the procedure for compound 9, but with 1 (139 mg, 0.40 mmol), 1,4-oxathiane (56 μL, 0.60 mmol) and HBF_4_·Et_2_O (55 μL, 0.40 mmol). Yield: 147 mg (0.30 mmol, 74%). Red solid. Anal. calc. for C_15_H_21_S_2_O_3_MoBF_4_: C: 36.31; H: 4.27; S: 12.92. Found: C: 36.10; H: 4.09; S: 12.69. Positive-ion MS (acetone): *m*/*z* (%) = 411 (100) [M]^+^. ^1^H NMR [500 MHz, CD_2_Cl_2_]: *δ* = 1.38 (t, ^3^*J*(^1^H,^1^H) = 7.4 Hz, 3H, SCH_2_C*H*_3_); 2.66–2.73 (m, 1H, C_5_H_4_C*H*_2_CH_2_S); 2.75–2.81 (m, 2H, CH_2_, SC*H*_2_CH_3_); 2.90–3.00 (m, 4H of OC_4_H_8_S, 1H of C_5_H_4_C*H*_2_CH_2_S); 3.70–3.76 (m, 1H, C_5_H_4_CH_2_C*H*_2_S); 3.84–3.92 (m, 1H, C_5_H_4_CH_2_C*H*_2_S); 3.96–4.04 (m, 4H, OC_4_H_8_S); 4.86–4.87 (m, 1H, C_5_H_4_); 5.58 (s, 1H, C_5_H_4_); 5.69 (s, 1H, C_5_H_4_); 6.38 (s, 1H, C_5_H_4_). ^13^C NMR [126 MHz, CD_2_Cl_2_]: *δ* = 13.7 (SCH_2_CH_3_); 26.0 (C_5_H_4_CH_2_CH_2_S); 34.1 (OC_4_H_8_S); 38.4 (SCH_2_CH_3_); 54.0 (C_5_H_4_CH_2_CH_2_S); 68.9 (OC_4_H_8_S); 82.6 (C_5_H_4_); 87.2 (C_5_H_4_); 89.2 (C_5_H_4_); 100.0 (C_5_H_4_); 142.5 (C_5_H_4_); 241.5 (CO); 243.5 (CO). IR (ATR, cm^−1^): 1986 *vs.* [*ν*_a_(CO)], 1885 *vs.* [*ν*_s_(CO)], 1026 *vs.* [*ν*(BF)]. Single crystals of 11-*cis* suitable for X-ray diffraction analysis were prepared by overlayering of the dichloromethane solution of 11 with diethyl ether.

### Synthesis of [{η^5^:κ*S*-C_5_H_5_(CH_2_)_2_SPh}Mo(CO)_2_(SMe_2_)][BF_4_] (12)

The steps of synthesis followed the procedure for compound 9, but with 2 (98 mg, 0.25 mmol), Me_2_S (37 μL, 0.50 mmol) and HBF_4_·Et_2_O (33 μL, 0.25 mmol). Yield: 98 mg (0.20 mmol, 78%). Red solid. Anal. calc. for C_17_H_19_S_2_O_2_MoBF_4_: C: 40.66; H: 3.81; S: 12.77. Found: C: 40.38; H: 3.57; S: 12.52. Positive-ion MS (acetone): *m*/*z* (%) = 355 (100) [M −  SC_2_H_6_]^+^. ^1^H NMR [400 MHz, CD_2_Cl_2_]: *δ* = 2.53 (s, 6H, (CH_3_)_2_S); 2.72–2.81 (m, 1H, C_5_H_4_C*H*_2_CH_2_S); 2.93–3.01 (m, 1H, C_5_H_4_C*H*_2_CH_2_S); 3.87–3.95 (m, 1H, C_5_H_4_CH_2_C*H*_2_S); 4.19–4.28 (m, 1H, C_5_H_4_CH_2_C*H*_2_S); 4.96 (s, 1H, C_5_H_4_); 5.56 (s, 1H, C_5_H_4_); 5.85 (s,1H, C_5_H_4_); 6.39 (s, 1H, C_5_H_4_); 7.50–7.58 (m, 5H, C_6_H_5_). ^13^C NMR [126 MHz, CD_2_Cl_2_]: *δ* = 27.0 (C_5_H_4_CH_2_CH_2_S); 28.3 ((CH_3_)_2_S); 61.3 (C_5_H_4_CH_2_CH_2_S); 82.8 (C_5_H_4_); 87.2 (C_5_H_4_); 89.5 (C_5_H_4_); 100.8 (C_5_H_4_); 130.6 (C_6_H_5_); 131.6 (C_6_H_5_); 131.8 (C_6_H_5_); 142.1 (C_5_H_4_); 240.2 (CO); 243.9 (CO). IR (ATR, cm^−1^): 1978 *vs.* [*ν*_a_(CO)], 1890 *vs.* [*ν*_s_(CO)], 1022 *vs.* [*ν*(BF)]. Single crystals of 12-*cis* suitable for X-ray diffraction analysis were prepared by the overlayering of dichloromethane solution of 12 with hexane.

### Synthesis of [{η^5^:κS-C_5_H_4_(CH_2_)_2_SPh}Mo(CO)_2_{S(CH_2_)_4_}][BF_4_] (13)

The steps of synthesis followed the procedure for compound 9, but with 2 (68 mg, 0.17 mmol), tetrahydrothiophene (22 μL, 0.25 mmol) and HBF_4_·Et_2_O (23 μL, 0.17 mmol). Yield: 80 mg (0.15 mmol, 89%). Red solid. Anal. calc. for C_19_H_21_S_2_O_2_MoBF_4_: C: 43.20; H: 4.01; S: 12.14. Found: C: 42.98; H: 3.83; S: 11.96. Positive-ion MS (acetone): *m*/*z* (%) = 443 (100) [M]^+^. ^1^H NMR [500 MHz, CD_2_Cl_2_, mixture 5 : 2 of isomers *cis* : *trans*]: *δ* = 2.07–2.21 (m, 4H, H^3,3,4,4^, C_4_H_8_S); 2.37–2.48 (m, 1H, C_5_H_4_C*H*_2_CH_2_S of *trans*); 2.62–2.71 (m, 1H, C_5_H_4_C*H*_2_CH_2_S of *trans*); 2.74–2.85 (m,1H, C_5_H_4_C*H*_2_CH_2_S of *cis*); 2.90–3.00 (m, 1H, C_5_H_4_C*H*_2_CH_2_S of *cis*); 3.17 (t, ^3^*J*(^1^H,^1^H) = 5.8 Hz, 4H, H^2,2,5,5^, C_4_H_8_S); 3.52–3.61 (m, 1H, C_5_H_4_CH_2_C*H*_2_S of *trans*); 3.73–3.80 (m, 1H, C_5_H_4_CH_2_C*H*_2_S of *trans*); 3.94 (dt, ^2^*J*(^1^H,^1^H) = 12.7 Hz, ^3^*J*(^1^H,^1^H) = 5.3 Hz, 1H, C_5_H_4_CH_2_C*H*_2_S of *cis*); 4.15–4.22 (m, 1H, C_5_H_4_CH_2_C*H*_2_S of *cis*); 4.97 (d, ^3^*J*(^1^H,^1^H) = 1.4 Hz, 1H, C_5_H_4_ of *cis*); 5.15 (s, 1H, C_5_H_4_ of *trans*); 5.24 (s, 1H, C_5_H_4_ of *trans*); 5.54 (s, 1H, C_5_H_4_ of *cis*); 5.87 (s, 1H, C_5_H_4_ of *cis*); 6.17 (s, 1H, C_5_H_4_ of *trans*); 6.35 (s, 1H, C_5_H_4_ of *cis*); 6.42 (s, 1H, C_5_H_4_ of *trans*); 7.49–7.54 (m, 5H, C_6_H_5_ of *cis*); 7.55–7.57 (m, 3H, C_6_H_5_ of *trans*); 7.63–7.66 (m, 2H, C_6_H_5_ of *trans*). ^13^C NMR [126 MHz, CD_2_Cl_2_]: *δ* = 26.7 (C_5_H_4_CH_2_CH_2_S); 31.0 (SC_4_H_8_); 31.2 (SC_4_H_8_); 44.4 (SC_4_H_8_); 45.0 (SC_4_H_8_); 61.5 (C_5_H_4_CH_2_CH_2_S); 83.3 (C_5_H_4_ of *cis*); 87.2 (C_5_H_4_ of *cis*); 89.1 (C_5_H_4_ of *cis*); 90.4 (C_5_H_4_ of *trans*); 92.7 (C_5_H_4_ of *trans*); 94.6 (C_5_H_4_ of *trans*); 95.2 (C_5_H_4_ of *trans*); 101.1 (C_5_H_4_ of *cis*); 130.5 (C_6_H_5_); 130.6 (C_6_H_5_); 131.9 (C_6_H_5_); 141.5 (C_5_H_4_); 231.3 (CO of *trans*); 232.3 (CO of *trans*); 241.3 (CO of *cis*); 244.8 (CO of *cis*). IR (ATR, cm^−1^): 1978 *vs.* [*ν*_a_(CO)], 1890 *vs.* [*ν*_s_(CO)], 1022 *vs.* [*ν*(BF)].

### Synthesis of [{η^5^:κ*S*-C_5_H_4_(CH_2_)_2_SPh}Mo(CO)_2_{κ*S*-S(CH_2_)_4_O}][BF_4_] (14)

The steps of synthesis followed the procedure for compound 9, but with 2 (198 mg, 0.50 mmol), 1,4-oxathiane (70 μL, 0.75 mmol) and HBF_4_·Et_2_O (67 μL, 0.50 mmol). Yield: 221 mg (0.41 mmol, 81%). Red solid. Anal. calc. for C_19_H_21_S_2_O_3_MoBF_4_: C: 41.93; H: 3.89; S: 11.78. Found: C: 41.67; H: 3.66; S: 11.57. Positive-ion MS (acetone): *m*/*z* (%) = 355 (100) [M −  OC_4_H_8_S]^+^. ^1^H NMR [500 MHz, CD_2_Cl_2_]: *δ* = 2.63–2.83 (m, 2H, C_5_H_4_C*H*_2_CH_2_S); 2.93–3.06 (m, 4H, OC_4_H_8_S); 3.86–3.96 (1H, C_5_H_4_CH_2_C*H*_2_S); 3.99 (s, 4H, OC_4_H_8_S); 4.22–4.30 (m, 1H, C_5_H_4_CH_2_C*H*_2_S); 4.94 (s, 1H, C_5_H_4_); 5.58 (s, 1H, C_5_H_4_); 5.86 (s, 1H, C_5_H_4_); 6.47 (s, 1H, C_5_H_4_); 7.49–7.58 (m, 5H, C_6_H_5_). ^13^C NMR [126 MHz, CD_2_Cl_2_]: *δ* = 27.0 (C_5_H_4_CH_2_CH_2_S); 38.6 (OC_4_H_8_S); 54.4 (C_5_H_4_CH_2_CH_2_S); 69.0 (OC_4_H_8_S); 82.8 (C_5_H_4_); 87.0 (C_5_H_4_); 89.4 (C_5_H_4_); 100.6 (C_5_H_4_); 130.6 (C_6_H_5_); 131.5 (C_6_H_5_); 131.6 (C_6_H_5_); 142.0 (C_5_H_4_); 240.1 (CO); 243.4 (CO). IR (ATR, cm^−1^): 1984 *vs.* [*ν*_a_(CO)], 1901 *vs.* [*ν*_s_(CO)], 1030 *vs.* [*ν*(BF)].

### Synthesis of [(η^5^-C_5_H_5_)Mo(CO)_2_(SMe_2_)_2_][BF_4_] (15)

The steps of synthesis followed the procedure for compound 9, but with [(η^5^-C_5_H_5_)Mo(CO)_2_(η^3^-C_3_H_5_)] (120 mg, 0.47 mmol), Me_2_S (104 μL, 1.41 mmol) and HBF_4_·Et_2_O (63 μL, 0.47 mmol). Yield: 157 mg (0.37 mmol, 78%). Red solid. Anal. calc. for C_11_H_17_S_2_O_2_MoBF_4_: C: 30.86; H: 4.00; S: 14.98. Found: C: 30.62; H: 3.77; S: 14.74. Positive-ion MS (acetone): *m*/*z* (%) = 335 (100) [M − 2 SC_2_H_6_ + 2 C_3_H_6_O]^+^. ^1^H NMR [500 MHz, CD_2_Cl_2_]: *δ* = 2.52 (s, 12H, (CH_3_)_2_S); 5.76 (s, 5H, C_5_H_5_). ^13^C NMR [126 MHz, CD_2_Cl_2_]: *δ* = 26.9 ((CH_3_)_2_S); 95.7 (C_5_H_5_); 244.9 (CO). IR (ATR, cm^−1^): 1982 *vs.* [*ν*_a_(CO)], 1888 *vs.* [*ν*_s_(CO)], 1029 *vs.* [*ν*(BF)]. Single crystals of 15-*trans* suitable for X-ray diffraction analysis were prepared by overlayering of the dichloromethane solution of 15 with diethyl ether.

### Synthesis of [(η^5^-C_5_H_5_)Mo(CO)_2_{(CH_2_)_4_S}_2_][BF_4_] (16)

The steps of synthesis followed the procedure for compound 9, but with [(η^5^-C_5_H_5_)Mo(CO)_2_(η^3^-C_3_H_5_)] (109 mg, 0.42 mmol), tetrahydrothiophene (93 μL, 1.05 mmol) and HBF_4_·Et_2_O (57 μL, 0.42 mmol)). Yield: 169 mg (0.35 mmol, 84%). Red solid. Anal. calc. for C_15_H_21_S_2_O_2_MoBF_4_: C: 37.52; H: 4.41; S: 13.35. Found: C: 37.25; H: 4.14; S: 13.11. Positive-ion MS (acetone): *m*/*z* (%) = 307 (100) [M −  SC_4_H_8_]^+^. ^1^H NMR [500 MHz, CD_2_Cl_2_]: *δ* = 2.15–2.18 (m, 4H, H^3,3,4,4^, C_4_H_8_S); 3.08–3.20 (m, 4H, H^2,2,5,5^, C_4_H_8_S); 5.72 (s, 5H, C_5_H_5_). ^13^C NMR [126 MHz, CD_2_Cl_2_]: *δ* = 31.2 (C^3,4^, C4H8S); 44.2 (C^2,5^, C_4_H_8_S); 95.8 (C_5_H_5_); 246.1 (CO). IR (ATR, cm^−1^): 1960 *vs.* [*ν*_a_(CO)], 1898 *vs.* [*ν*_s_(CO)], 1027 *vs.* [*ν*(BF)]. Single crystals of 16-*cis* suitable for X-ray diffraction analysis were prepared by overlayering of the dichloromethane solution of 16 with diethyl ether.

### Synthesis of [(η^5^-C_5_H_5_)Mo(CO)_2_{κ*S*-S(CH_2_)_4_O}_2_][BF_4_] (17)

The steps of synthesis followed the procedure for compound 9, but with [(η^5^-C_5_H_5_)Mo(CO)_2_(η^3^-C_3_H_5_)] (180 mg, 0.70 mmol), 1,4-oxathiane (164 μL, 1.75 mmol) and HBF_4_·Et_2_O (95 μL, 0.70 mmol). Yield: 295 mg (0.58 mmol, 82%). Red solid. Anal. calc. for C_15_H_21_S_2_O_4_MoBF_4_: C: 35.17; H: 4.13; S: 12.52. Found: C: 34.93; H: 3.89; S: 12.30. Positive-ion MS (acetone): *m*/*z* (%) = 295 (100) [M −  OC_4_H_8_S − CO]^+^. ^1^H NMR [500 MHz, CD_2_Cl_2_]: *δ* = 2.79–2.83 (m, 4H, OC_4_H_8_S); 3.00–3.04 (m, 4H, OC_4_H_8_S); 4.01–4.04 (m, 8H, OC_4_H_8_S); 5.79 (s, 5H, C_5_H_5_). ^13^C NMR [126 MHz, CD_2_Cl_2_]: *δ* = 37.6 (OC_4_H_8_S); 69.0 (OC_4_H_8_S); 95.7 (C_5_H_5_); 243.9 (CO). IR (ATR, cm^−1^): 1981 *vs.* [*ν*_a_(CO)], 1903 *vs.* [*ν*_s_(CO)], 1049 *vs.* [*ν*(BF)].

### Synthesis of [{η^5^:κ*S*-C_5_H_4_(CH_2_)_2_SEt}Mo(CO)_2_(NCMe)][BF_4_] (18)

The steps of synthesis followed the procedure for compound 7, but with 1 (391 mg, 1.13 mmol) and HBF_4_·Et_2_O (152 μL, 1.13 mmol). Yield: 454 mg (1.05 mmol, 93%). Red solid. Anal. calc. for C_13_H_16_SO_2_NMoBF_4_: C: 36.05; H: 3.72; N: 3.23; S: 7.40. Found: C: 35.88; H: 3.47; N: 2.98; S: 7.17. Positive-ion MS (acetone): *m*/*z* (%) = 365 (100) [M −  MeCN + C_3_H_6_O]^+^. ^1^H NMR [500 MHz, CD_2_Cl_2_]: *δ* = 1.39 (t, ^3^*J*(^1^H,^1^H) = 7.2 Hz, 3H, SCH_2_C*H*_3_); 2.54 (s, 3H, CH_3_CN); 2.59–2.66 (m, 1H, C_5_H_4_C*H*_2_CH_2_S); 2.81–2.95 (m, 2H, SC*H*_2_CH_3_); 2.93–2.99 (m, 1H, C_5_H_4_C*H*_2_CH_2_S); 3.65–3.71 (m, 1H, C_5_H_4_CH_2_C*H*_2_S); 4.04–4.12 (m, 1H, C_5_H_4_CH_2_C*H*_2_S); 4.82 (s, 1H, C_5_H_4_); 5.44 (s, 1H, C_5_H_4_); 5.76 (s, 1H, C_5_H_4_); 6.52 (s, 1H, C_5_H_4_). ^13^C NMR [126 MHz, CD_2_Cl_2_]: *δ* = 5.5 (CH_3_, CH_3_CN); 13.7 (SCH_2_CH_3_); 25.9 (C_5_H_4_CH_2_CH_2_S); 33.4 (SCH_2_CH3); 53.9 (C_5_H_4_CH_2_CH_2_S); 83.6 (C_5_H_4_); 87.8 (C_5_H_4_); 88.0 (C_5_H_4_); 104.9 (C_5_H_4_); 142.2 (C_5_H_4_); 144.7 (CN, CH_3_CN); 244.2 (CO); 247.8 (CO). IR (ATR, cm^−1^): 1994 *vs.* [*ν*_a_(CO)], 1886 *vs.* [*ν*_s_(CO)], 1054 *vs.* [*ν*(BF)]. Single crystals of 18 suitable for X-ray diffraction analysis were prepared by overlayering of the dichloromethane solution of 18 with hexane.

### Synthesis of [(η^5^-C_5_H_5_)Mo(CO)_2_(MeCN)_2_](CF_3_SO_3_) (19)

The steps of synthesis followed the procedure for compound 7, but with [(η^5^-C_5_H_5_)Mo(CO)_2_(η^3^-C_3_H_5_)] (109 mg, 0.42 mmol) and CF_3_SO_3_H (37 μL, 0.42 mmol). Yield: 150 mg (0.33 mmol, 80%). Red solid. Anal. calc. for C_12_H_11_N_2_SO_5_MoF_3_: C: 32.16; H: 2.47; N: 6.25; S: 7.15. Found: C: 31.93; H: 2.22; N: 6.02; S: 6.87. ^1^H NMR [400 MHz, CD_2_Cl_2_, 6 : 5 mixture of complexes [(η^5^-C_5_H_5_)Mo(CO)_2_(MeCN)_2_](CF_3_SO_3_) and [(η^5^-C_5_H_5_)Mo(CO)_2_(MeCN)(CF_3_SO_3_)] (a : b)]: *δ* = 1.97 (s, 3H, free CH_3_CN); 2.50 (s, 3H, CH_3_CN of b); 2.55 (s, 6H, CH_3_CN of a); 5.74 (s, 5H, C_5_H_5_ of b); 5.75 (s, 5H, C_5_H_5_ of a). IR (ATR, cm^−1^): 1990 *vs.* [*ν*_a_(CO)], 1882 *vs.* [*ν*_s_(CO)], 1261 *vs.* [*ν*_a_(SO_3_)], 1224 m [*ν*_s_(CF_3_)], 1151 s [*ν*_a_(CO)], 1032 s [*ν*_s_(SO_3_)]. Single crystals of 19 suitable for X-ray diffraction analysis were prepared by overlayering of the acetonitrile solution of 19 with diethyl ether.

## Author contributions

The authors contributed equally.

## Conflicts of interest

There are no conflicts to declare.

## Supplementary Material

RA-013-D3RA03383J-s001

RA-013-D3RA03383J-s002
