# Supplementary material for: Stabilization of propene molybdenum and tungsten half–sandwich complexes by intramolecular coordination of a thioether function
Source: RSC Adv. 2023 Jun 30;13(29):19746–56. doi: 10.1039/d3ra03383j (PMC10311468; doi:10.1039/d3ra03383j)
Supplement: RA-013-D3RA03383J-s001 [file RA-013-D3RA03383J-s001.pdf]

Electronic Supplementary Material (ESI) for RSC Advances.
This journal is © The Royal Society of Chemistry 2023

Supporting Information for:

Stabilization of propene complexes by intramolecular coordination of thioether function

Lukáš Hanzl,[a] Jaromír Vinklárek, [a] Libor Dostál,[a] Ivana Císařová,[b] Miroslava Litecká [c] and Jan Honzíček *[d]

[a]Department of General and Inorganic Chemistry, Faculty of Chemical Technology, University of Pardubice, Studentská 573, 532 10 Pardubice, Czech Republic.

[b]Department of Inorganic Chemistry, Faculty of Science, Charles University in Prague, Hlavova 2030/8, 128 43 Prague 2, Czech Republic.

[c]Department of Materials Chemistry, Institute of Inorganic Chemistry of the CAS, Husinec-Řež 1001, 25068 Řež, Czech Republic.

[d]Institute of Chemistry and Technology of Macromolecular Materials, Faculty of Chemical Technology, University of Pardubice, Studentská 573, 532 10 Pardubice, Czech Republic.

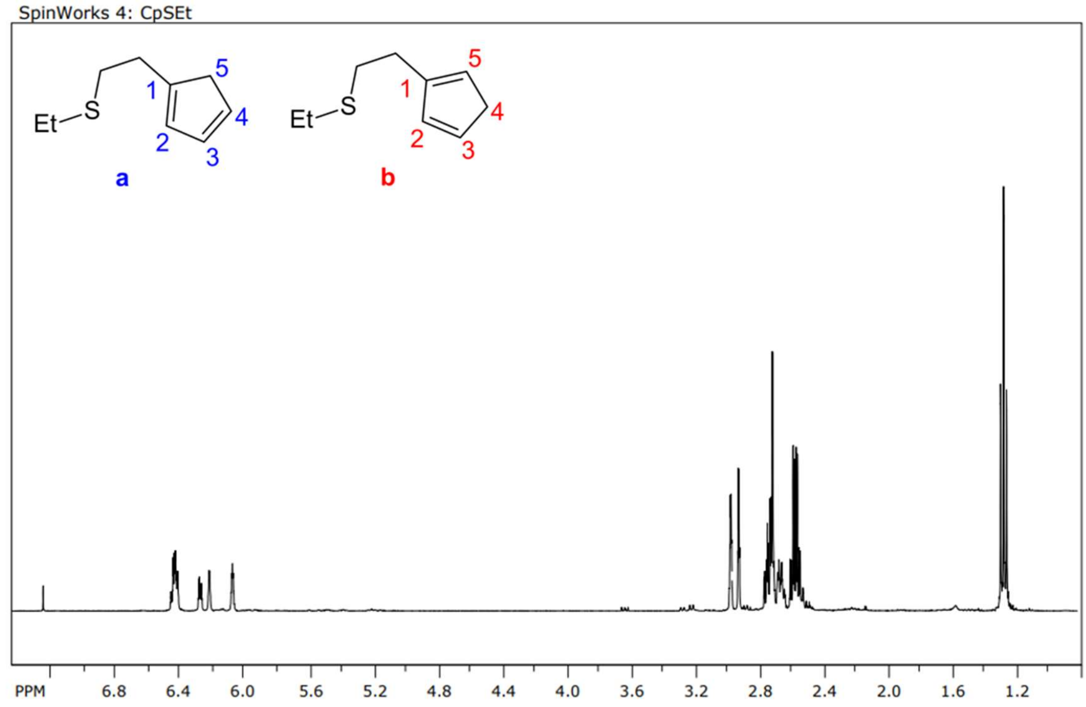

Figure S1: ^{1}H NMR spectrum of $C_5H_4(CH_2)_2SEt$ in $CDCl_3$ (400 MHz)

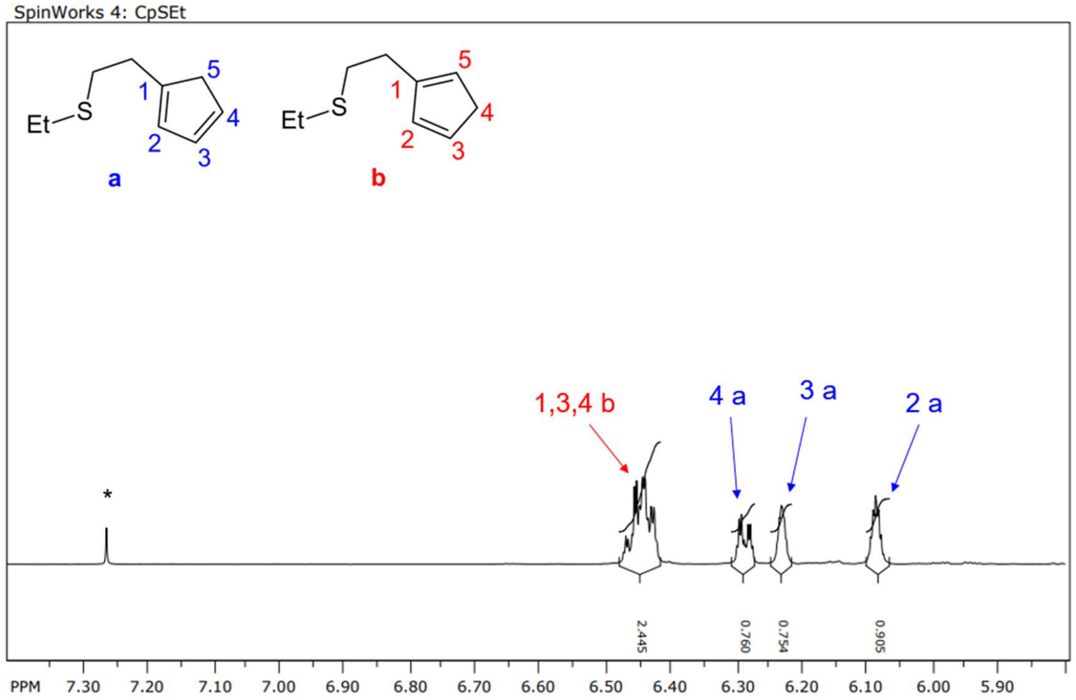

Figure S2: ^{1}H NMR spectrum of $C_5H_4(CH_2)_2SEt$ in $CDCl_3$ (400 MHz), close-up of the aromatic section

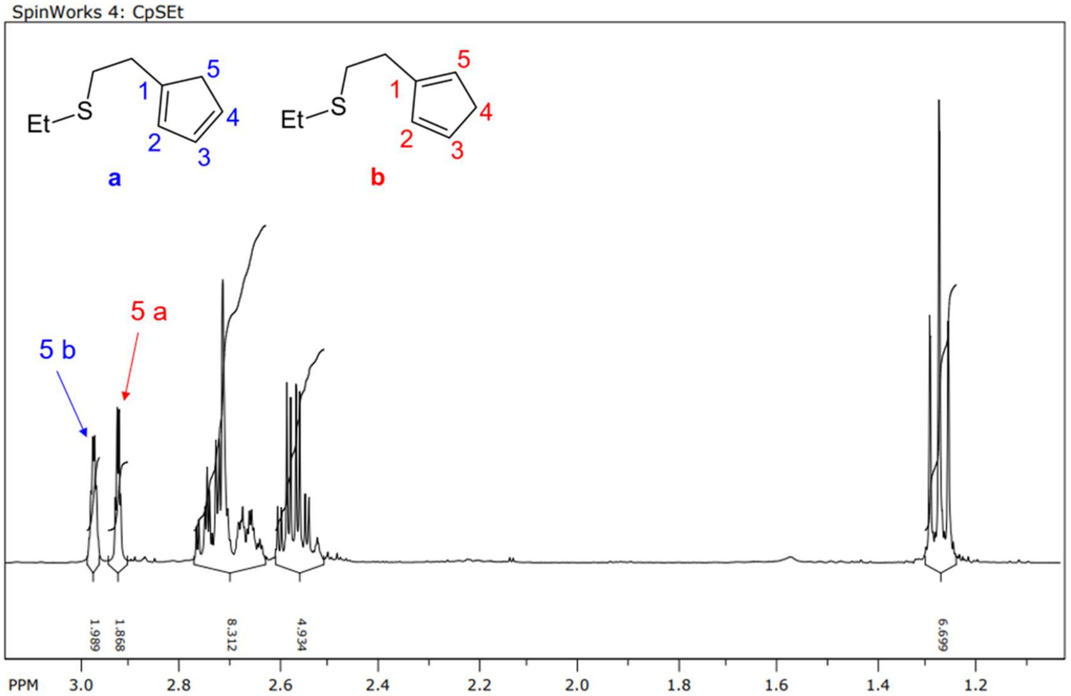

Figure S3: ^{1}H NMR spectrum of $C_5H_4(CH_2)_2SEt$ in $CDCl_3$ (400 MHz), close-up of the aliphatic section

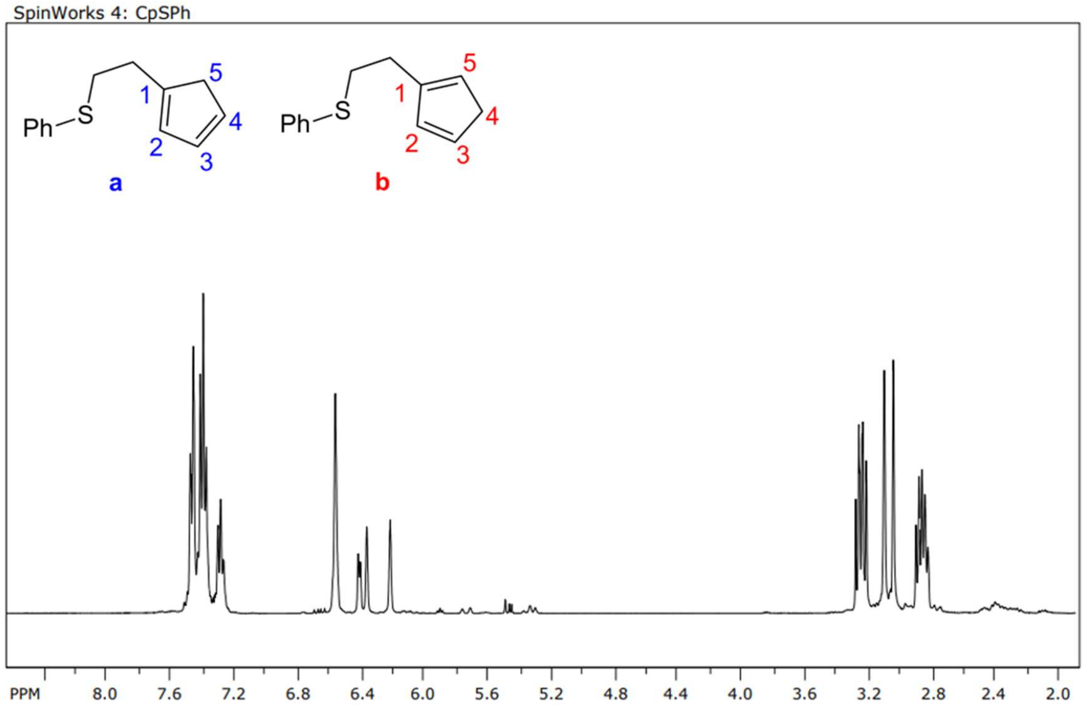

Figure S4: ^{1}H NMR spectrum of $C_5H_4(CH_2)_2SPh$ in $CDCl_3$ (400 MHz)

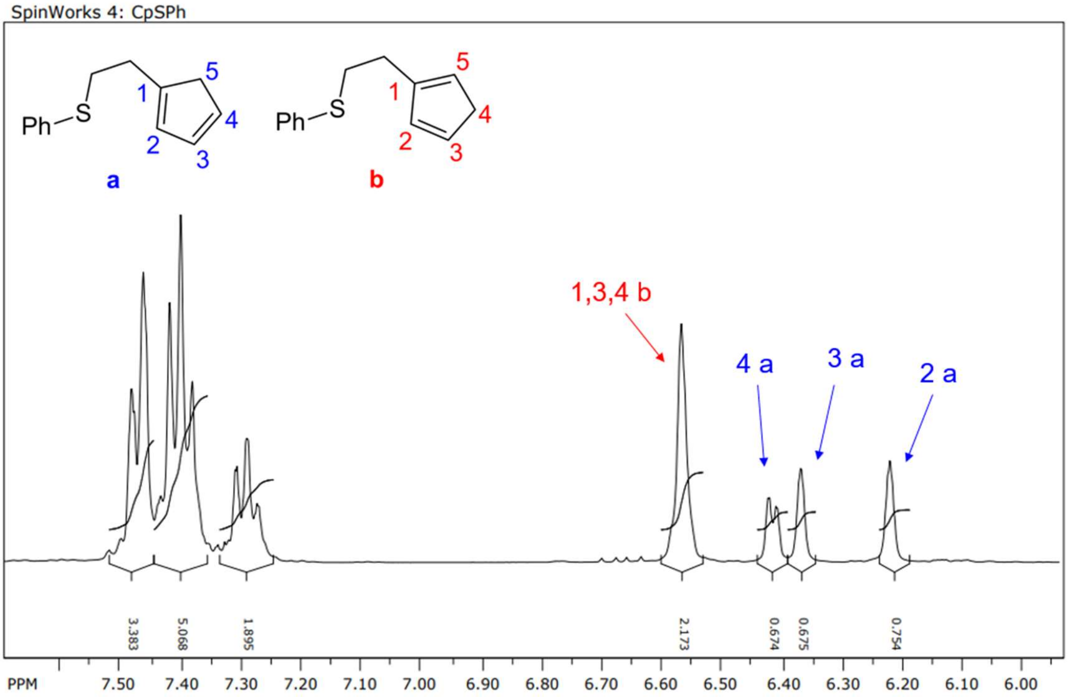

Figure S5: ^{1}H NMR spectrum of $C_5H_4(CH_2)_2SPh$ in $CDCl_3$ (400 MHz), close-up of the aromatic section

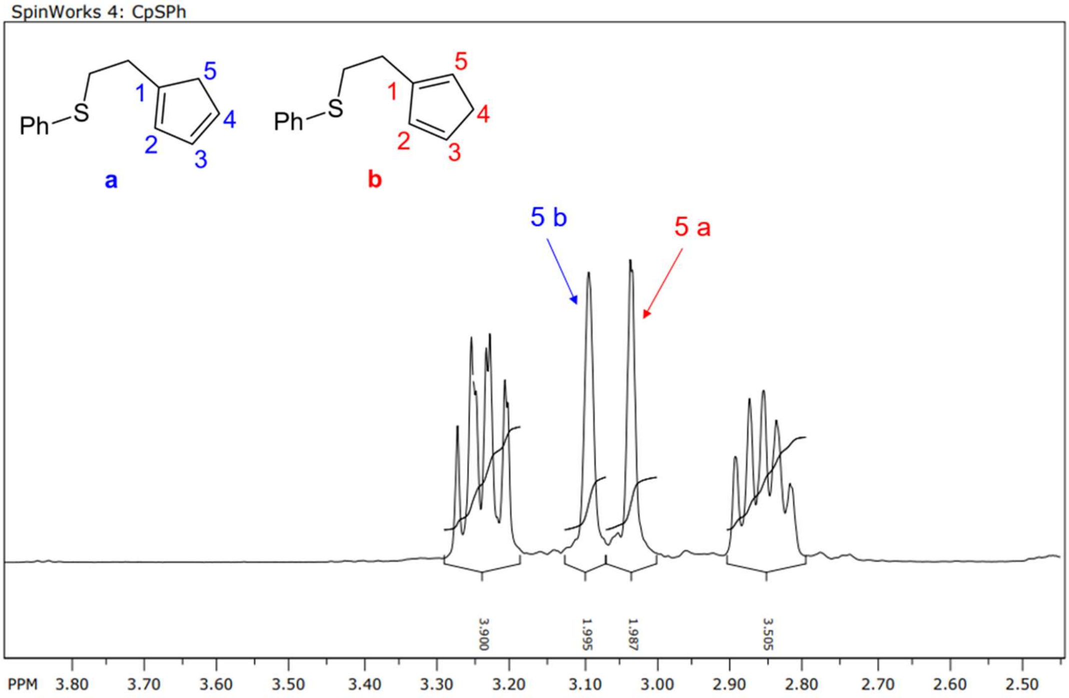

Figure S6: ^{1}H NMR spectrum of $C_5H_4(CH_2)_2SPh$ in $CDCl_3$ (400 MHz), close-up of the aliphatic section

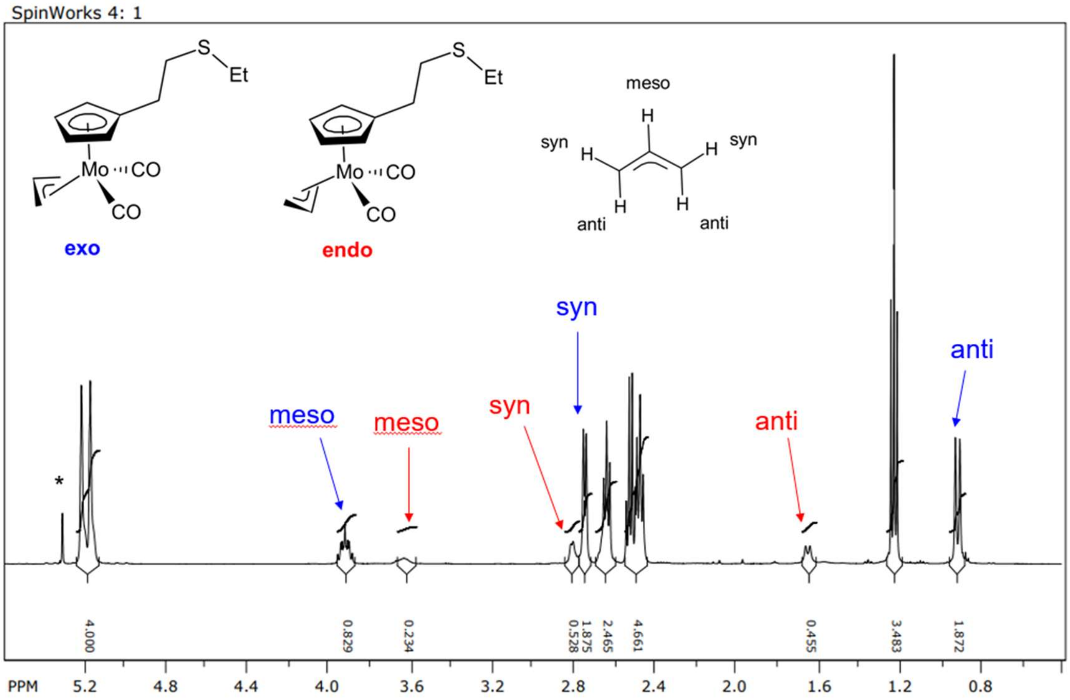

Figure S7: ^{1}H NMR spectrum of **1** in CD_2Cl_2 (500 MHz)

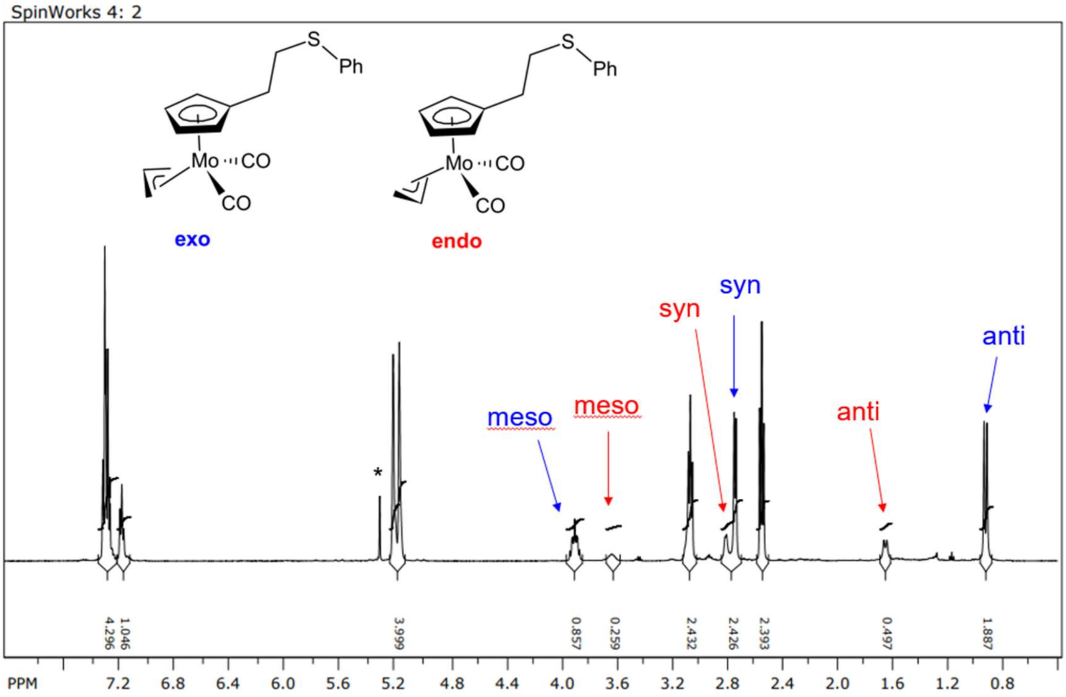

Figure S8: ^{1}H NMR spectrum of **2** in CD_2Cl_2 (500 MHz)

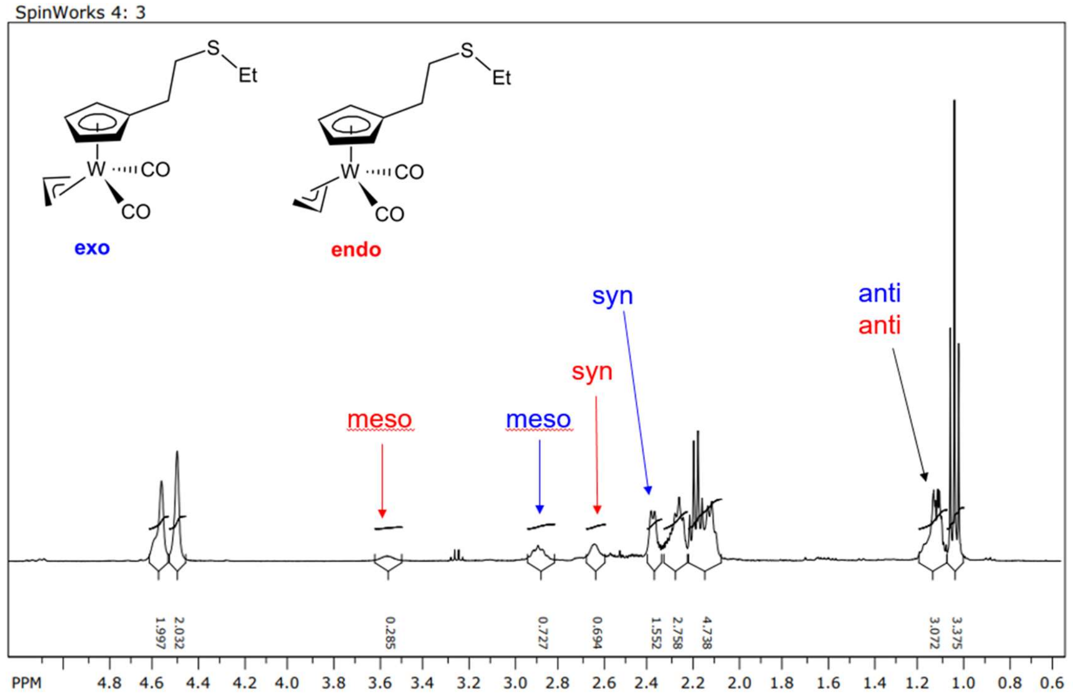

Figure S9: ^{1}H NMR spectrum of **3** in C_6D_6 (400 MHz)

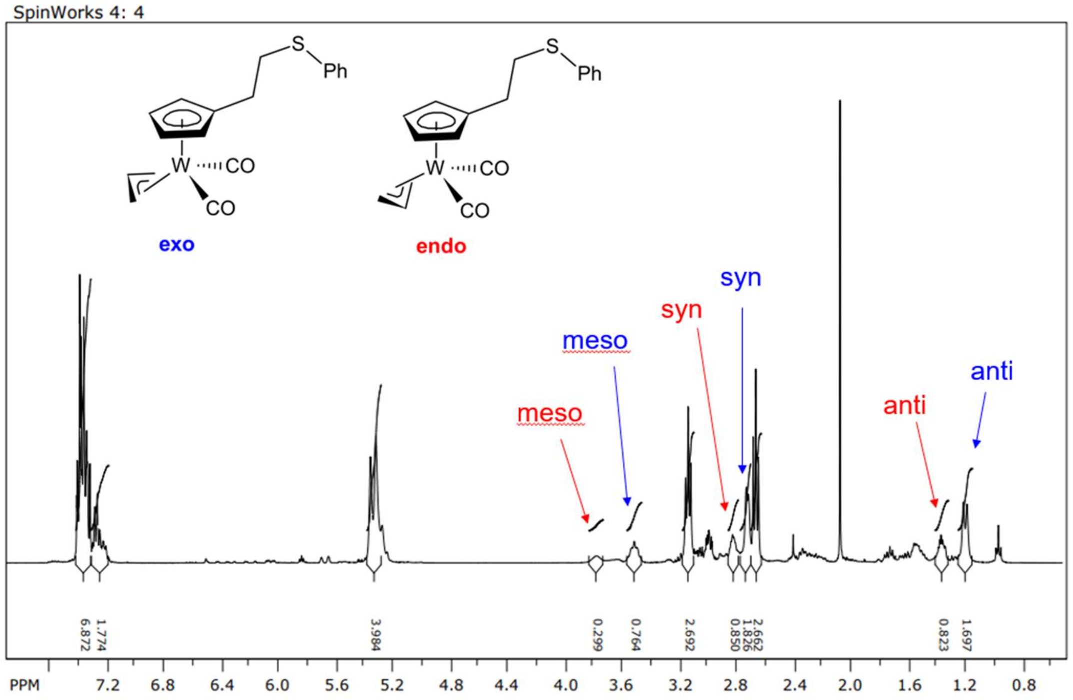

Figure S10: ^{1}H NMR spectrum of **4** in $CDCl_3$ (400 MHz)

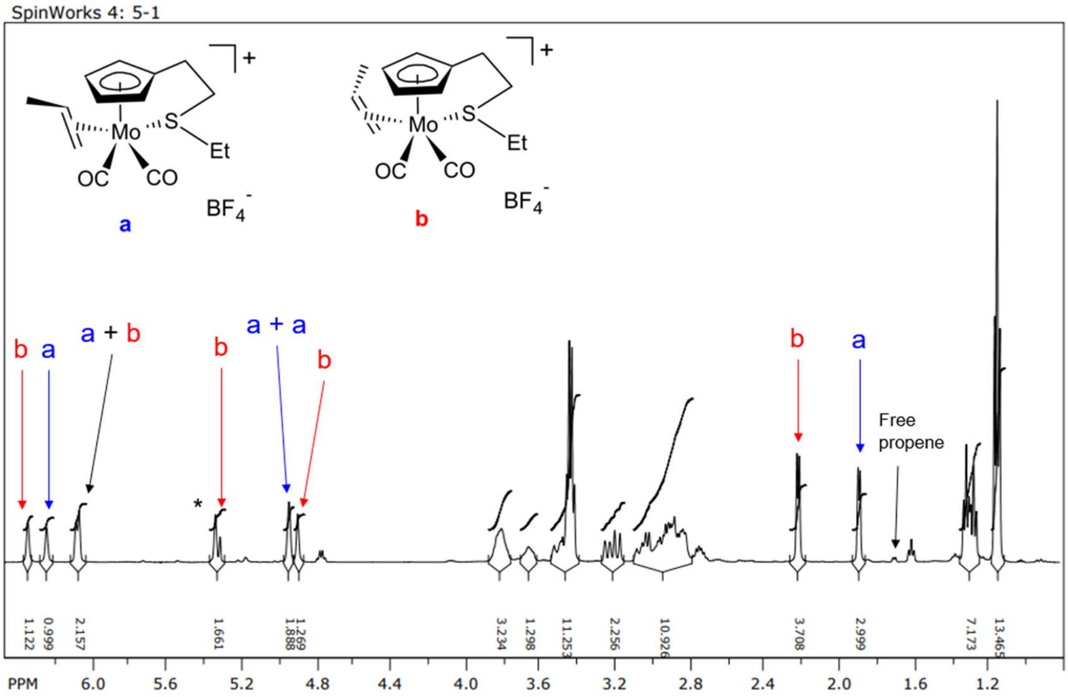

Figure S11: ^{1}H NMR spectrum of **5** in CD_2Cl_2 (500 MHz) measured directly after the preparation of the sample.

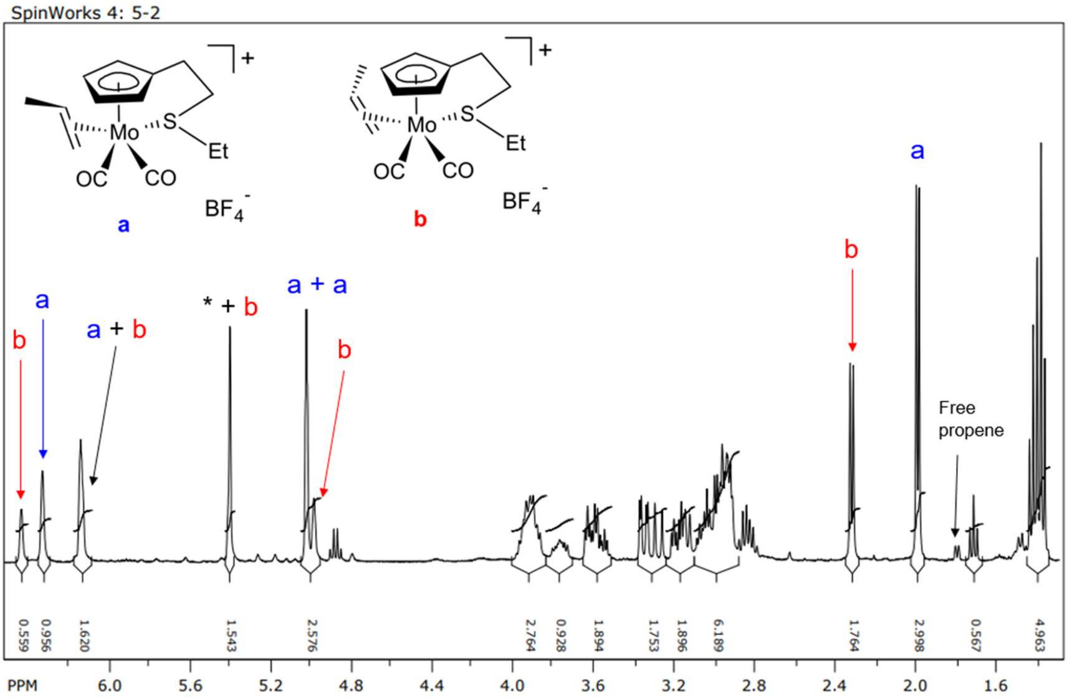

Figure S12: ^{1}H NMR spectrum of **5** in CD_2Cl_2 (500 MHz) measured after preparation with the internal standard.

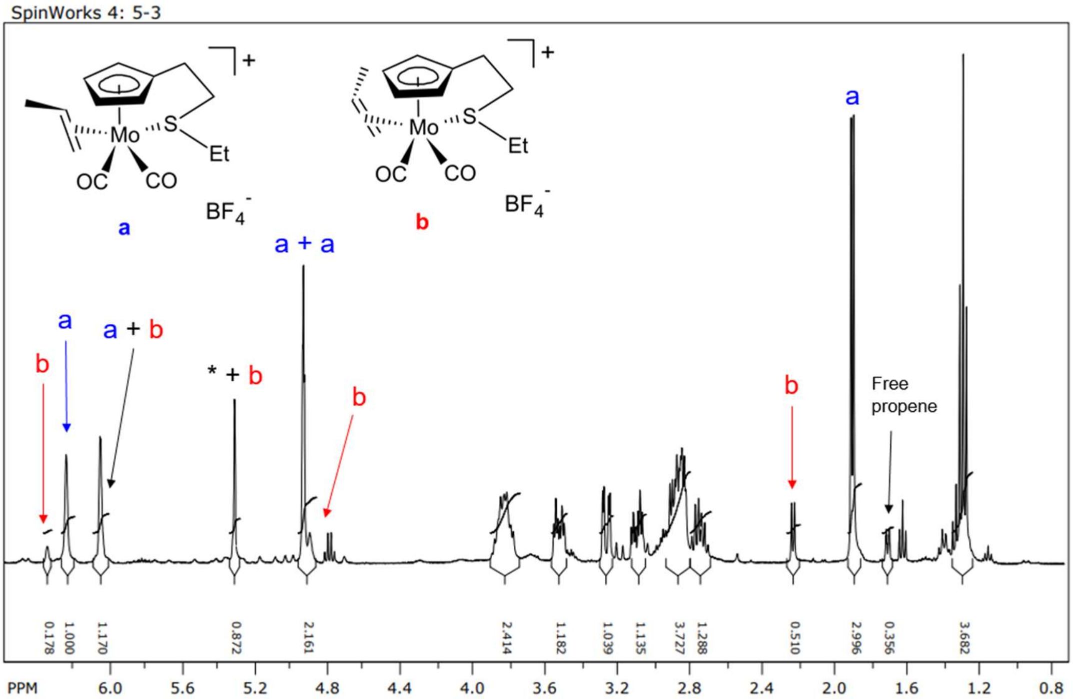

Figure S13: ^{1}H NMR spectrum of **5** in CD_2Cl_2 (500 MHz) measured after sitting at room temperature for two hours with the internal standard measured.

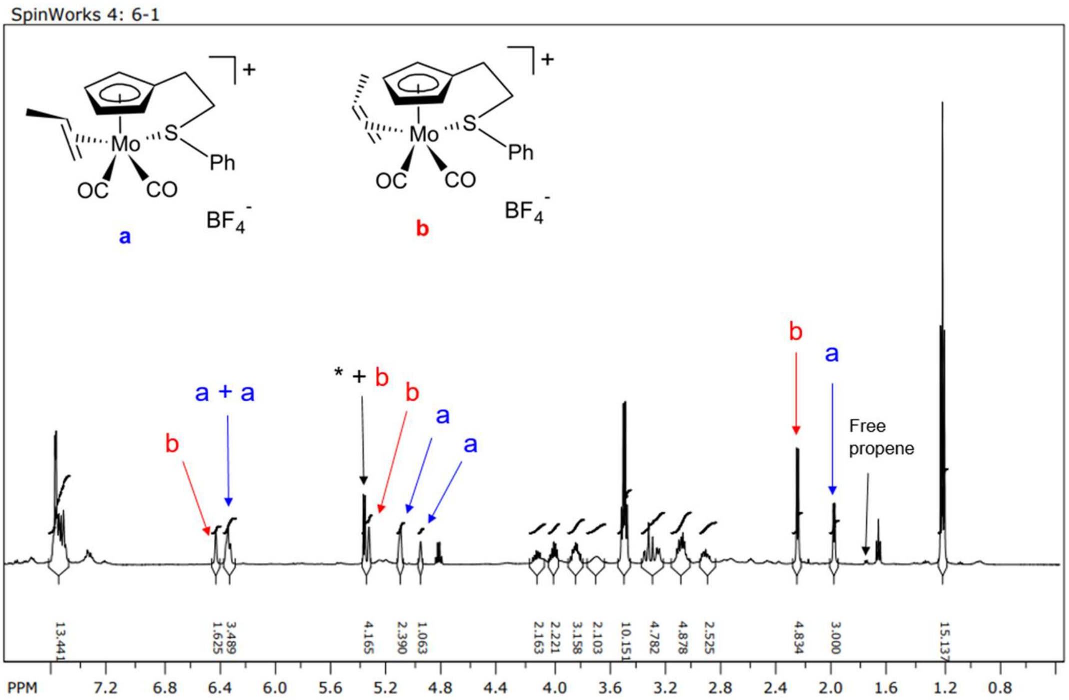

Figure S14: ^{1}H NMR spectrum of **6** in CD_2Cl_2 (500 MHz) measured directly after the preparation of the sample with the internal standard.

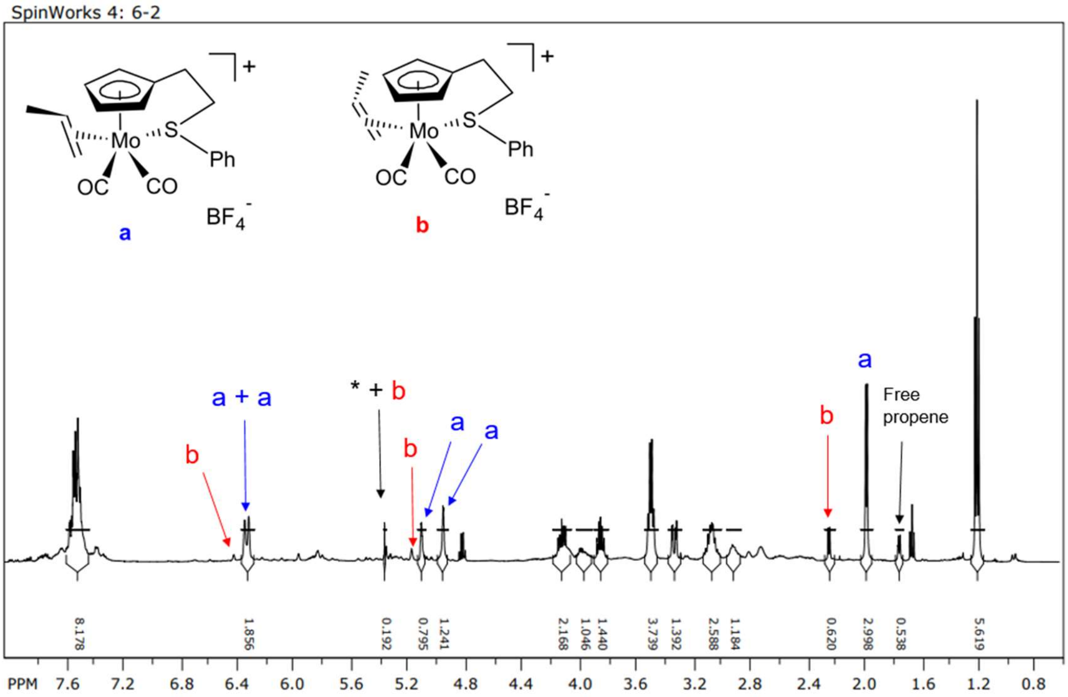

Figure S15: ^{1}H NMR spectrum of **6** in CD_2Cl_2 (500 MHz) measured after sitting at room temperature for two hours with the internal standard.

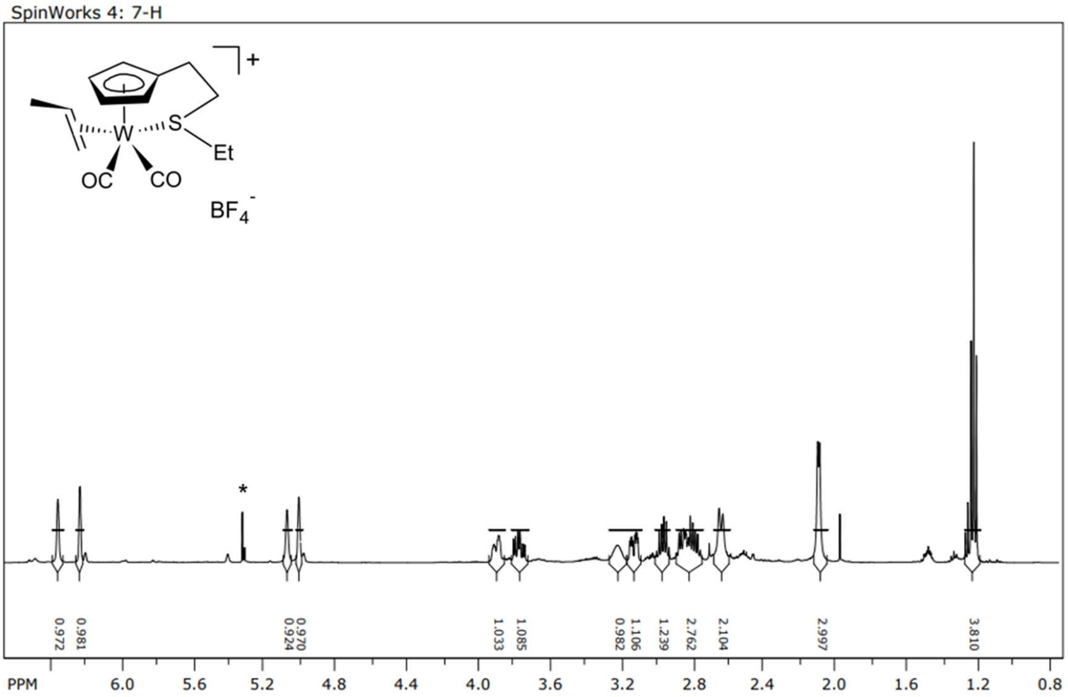

Figure S16: ^{1}H NMR spectrum of **7** in CD_2Cl_2 (500 MHz).

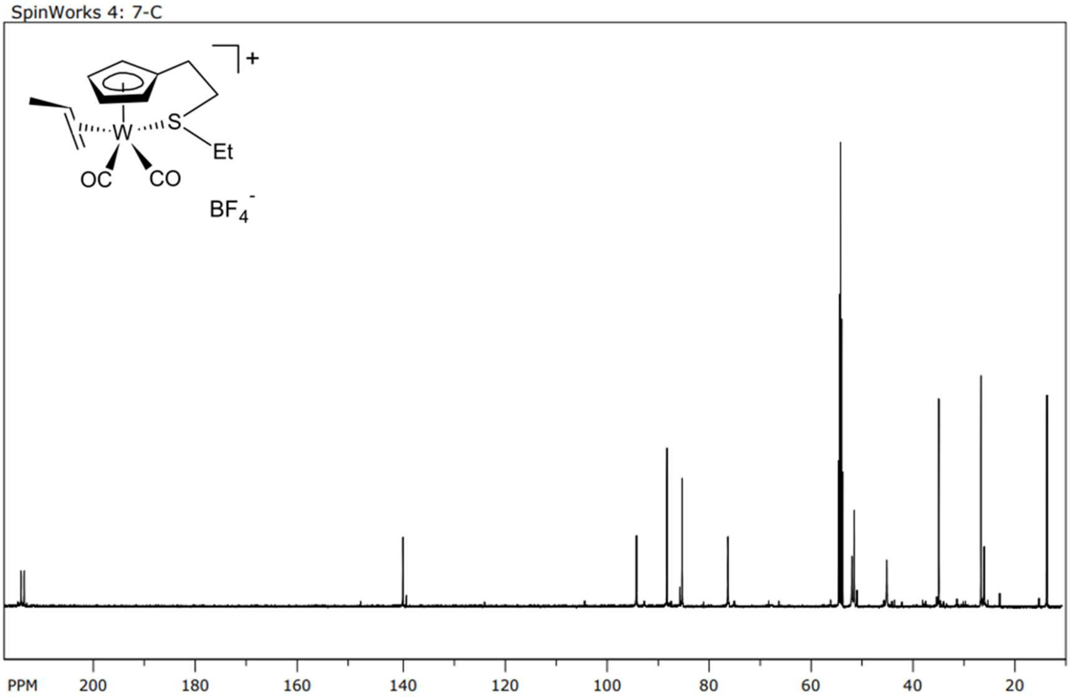

Figure S17: $^{13}C\{^{1}H\}$ NMR spectrum of **7** in CD_2Cl_2 (126 MHz).

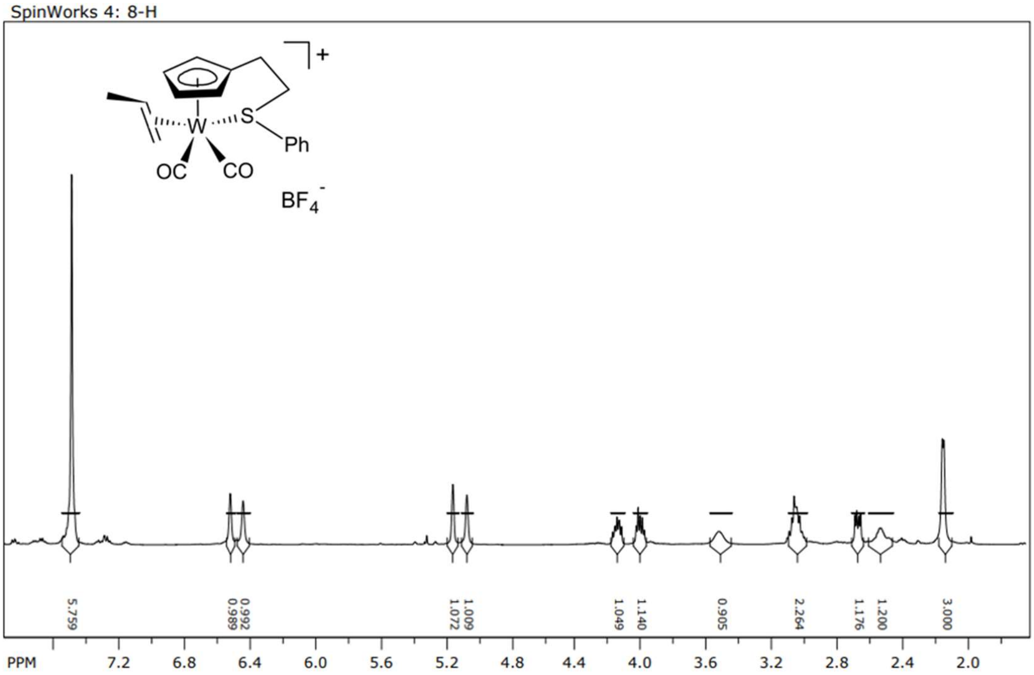

Figure S18: ^{1}H NMR spectrum of **8** in CD_2Cl_2 (500 MHz).

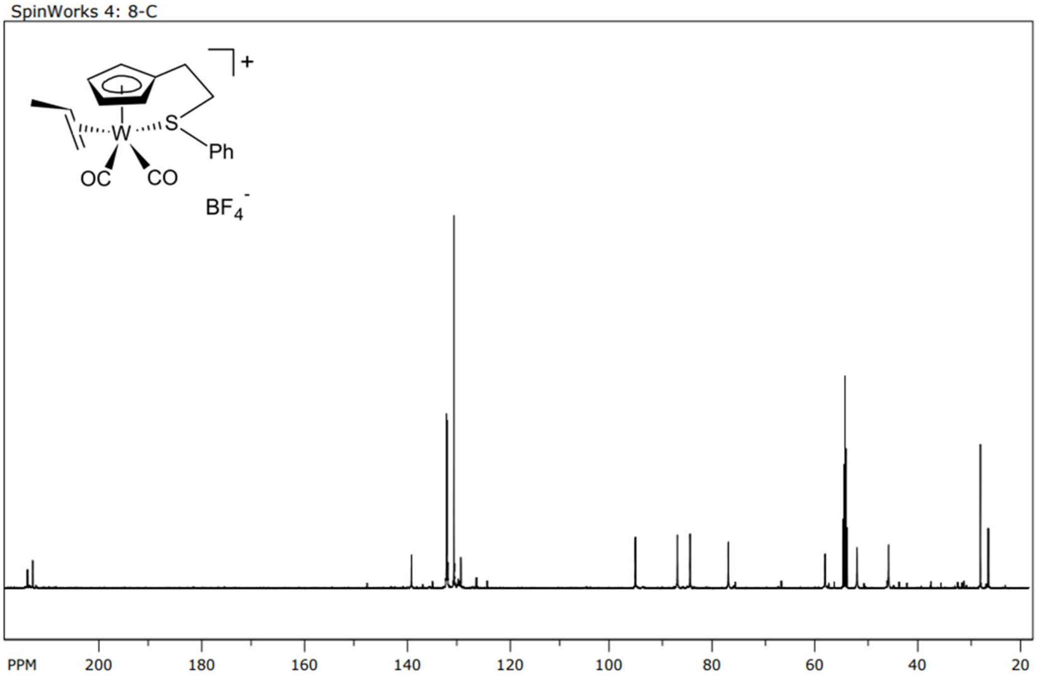

Figure S19: $^{13}C\{^1H\}$ NMR spectrum of **8** in CD_2Cl_2 (126 MHz).

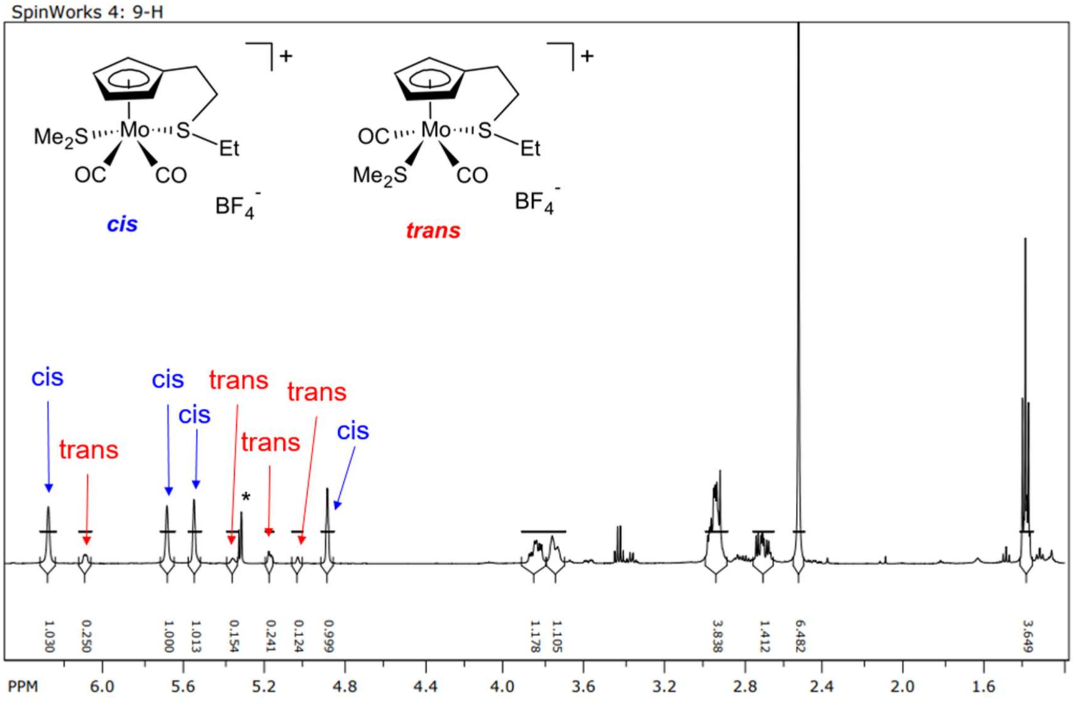

Figure S20: ^{1}H NMR spectrum of **9** in CD_2Cl_2 (500 MHz).

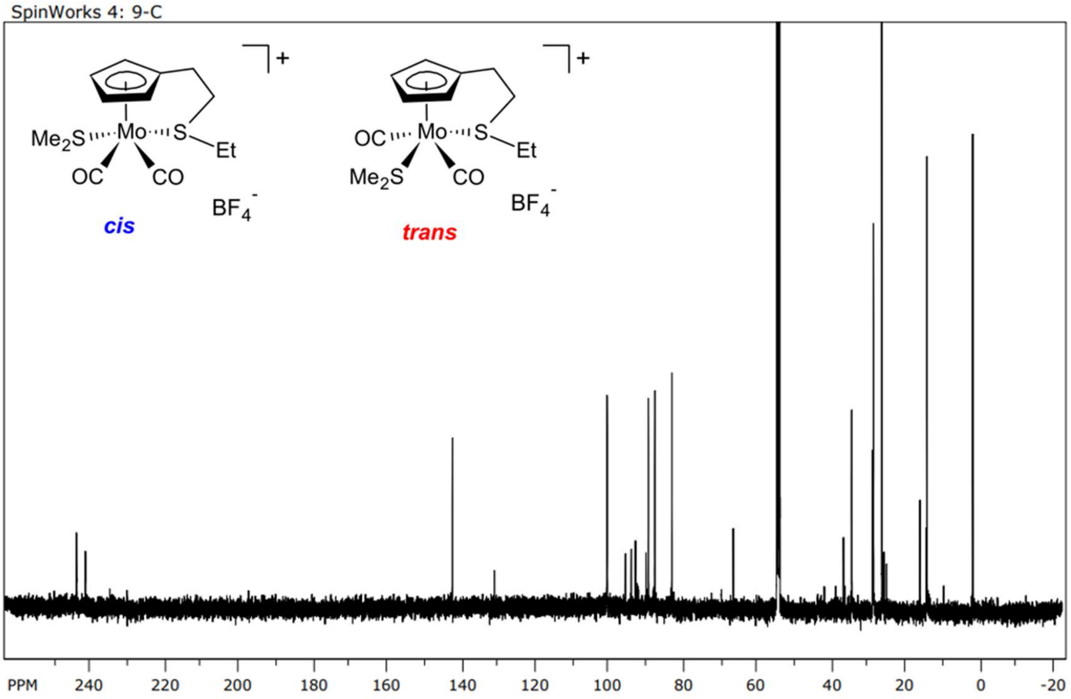

Figure S21: $^{13}C\{^{1}H\}$ NMR spectrum of **9** in CD_2Cl_2 (126 MHz).

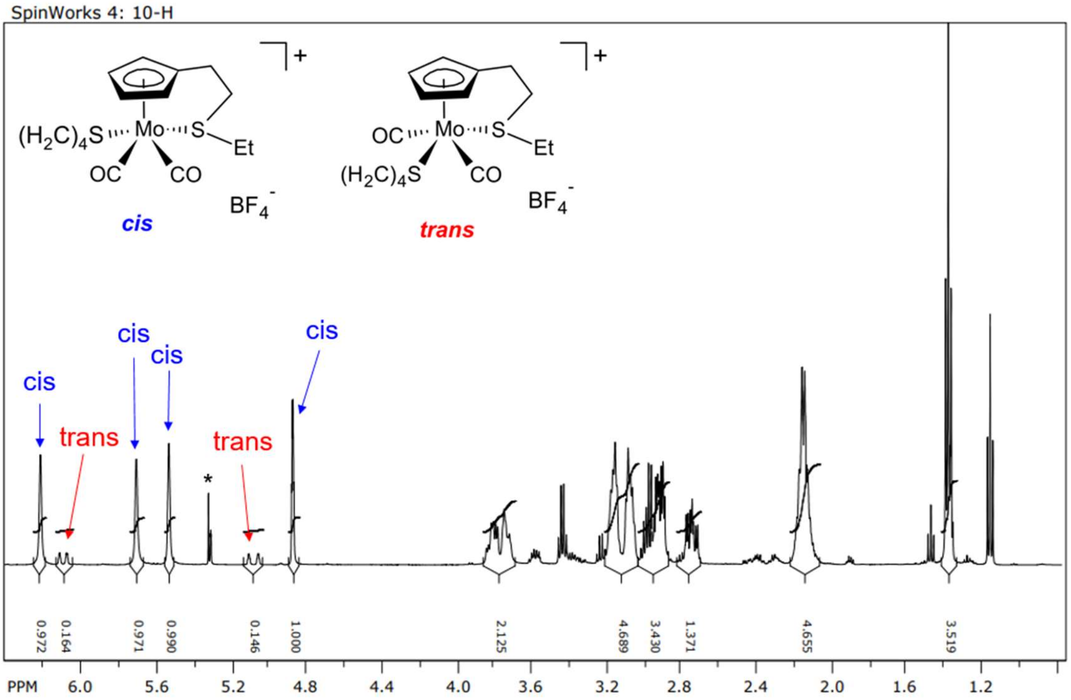

Figure S22: ^{1}H NMR spectrum of **10** in CD_2Cl_2 (500 MHz).

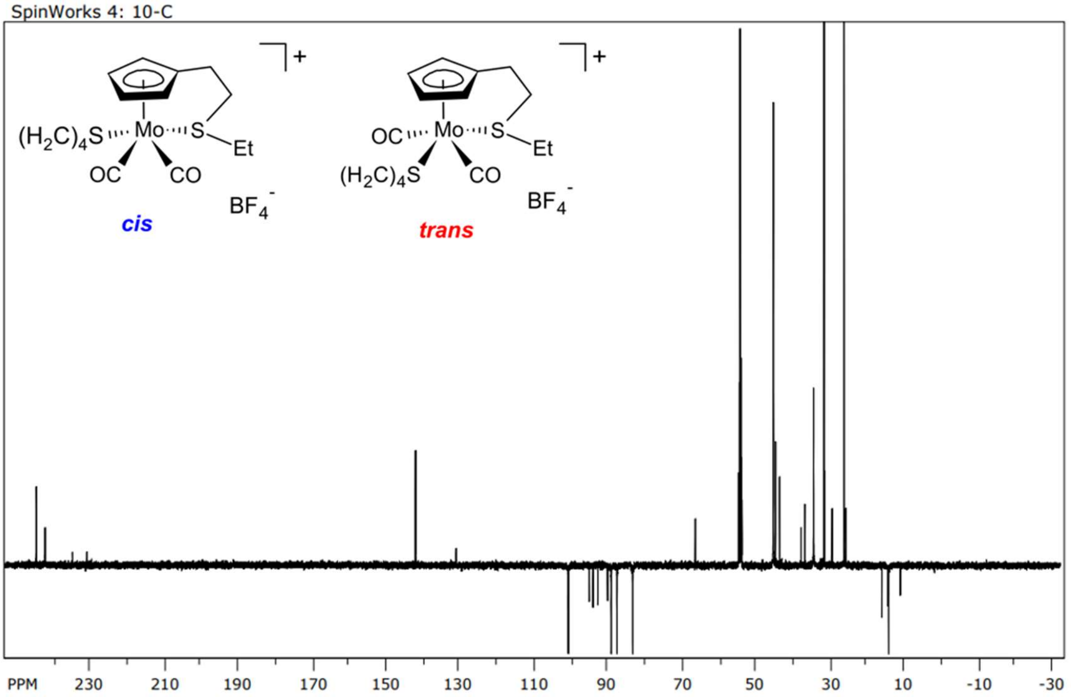

Figure 23: ^{13}C APT NMR spectrum of **10** in CD_2Cl_2 (126 MHz).

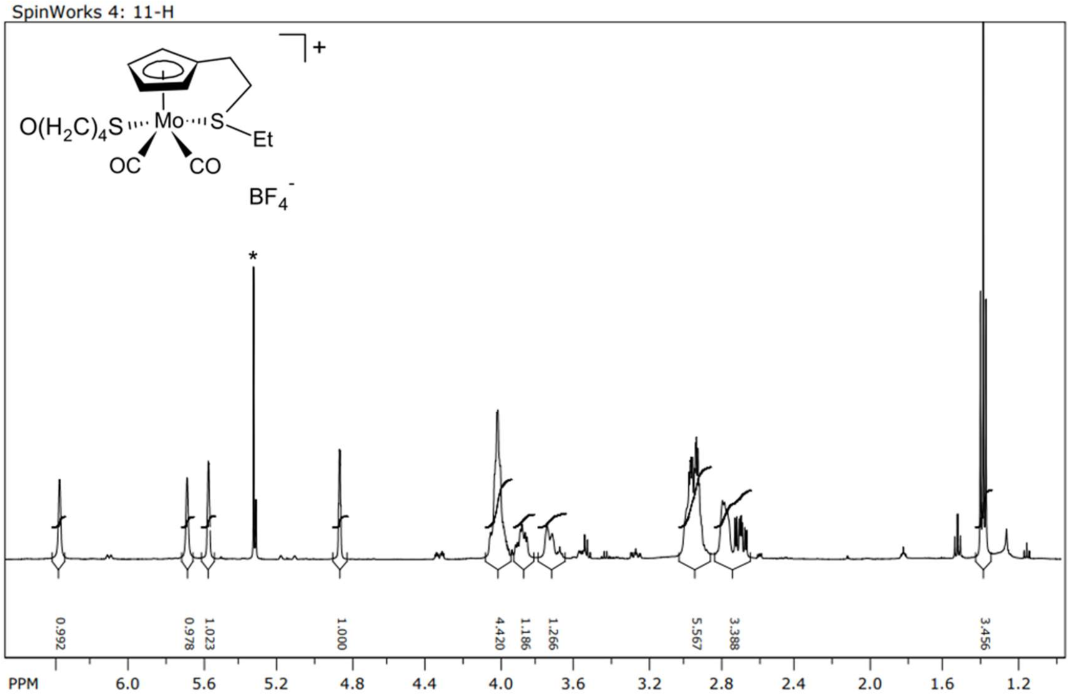

Figure S24: ^{1}H NMR spectrum of **11** in CD_2Cl_2 (500 MHz).

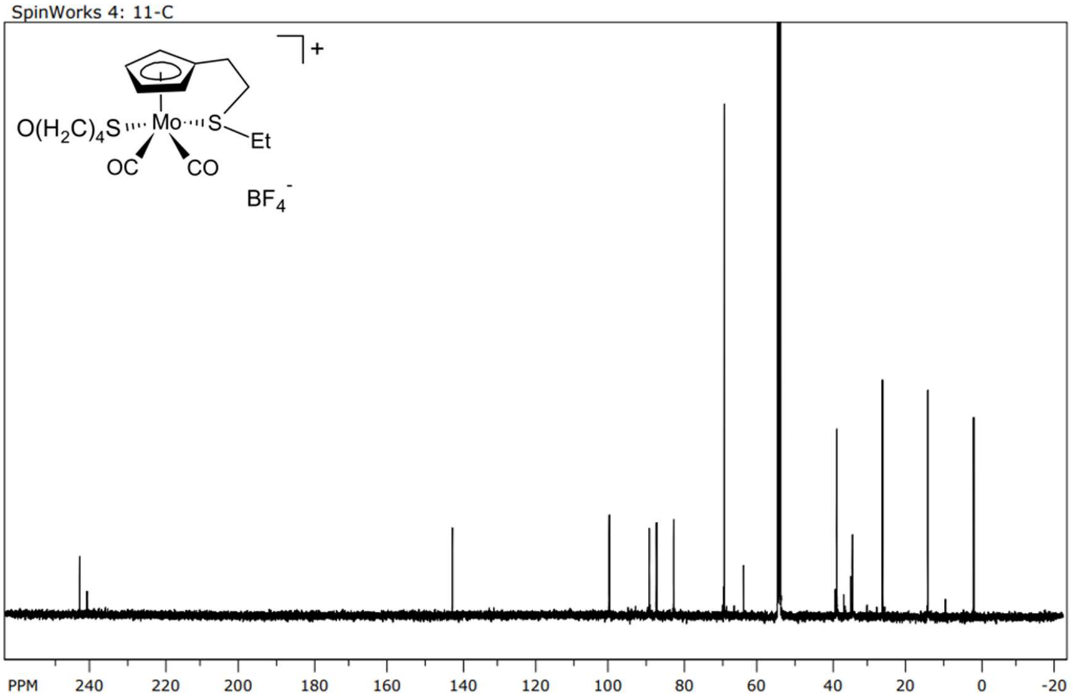

Figure S25: $^{13}C\{^{1}H\}$ NMR spectrum of **11** in CD_2Cl_2 (126 MHz).

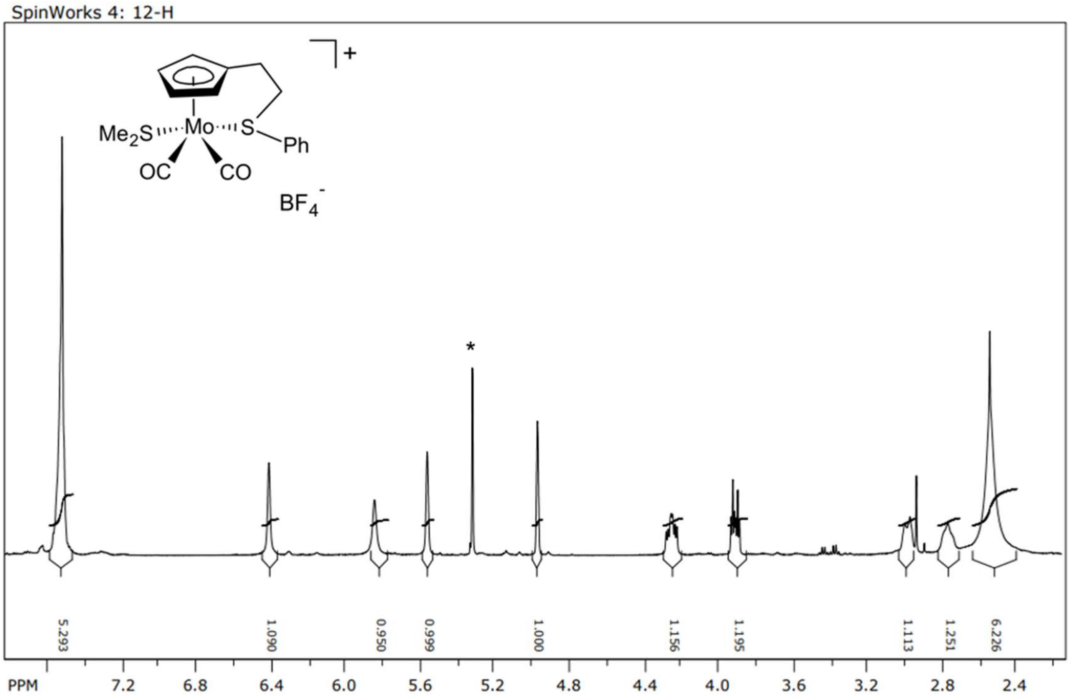

Figure S26: ^{1}H NMR spectrum of **12** in CD_2Cl_2 (400 MHz).

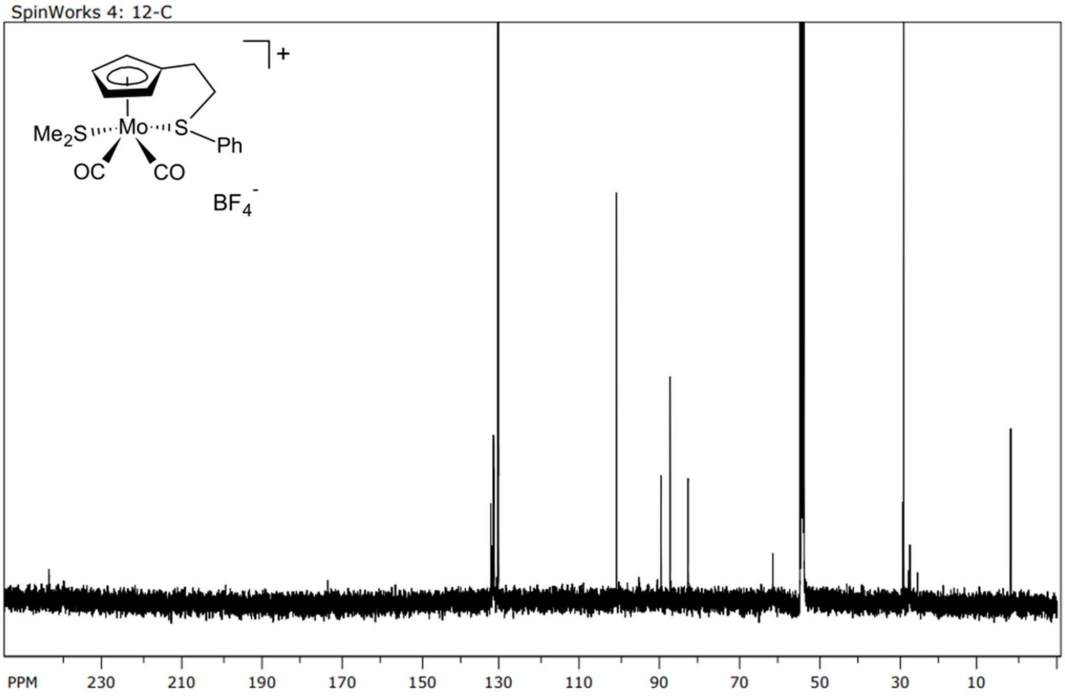

Figure S27: $^{13}C\{^{1}H\}$ NMR spectrum of **12** in CD_2Cl_2 (126 MHz).

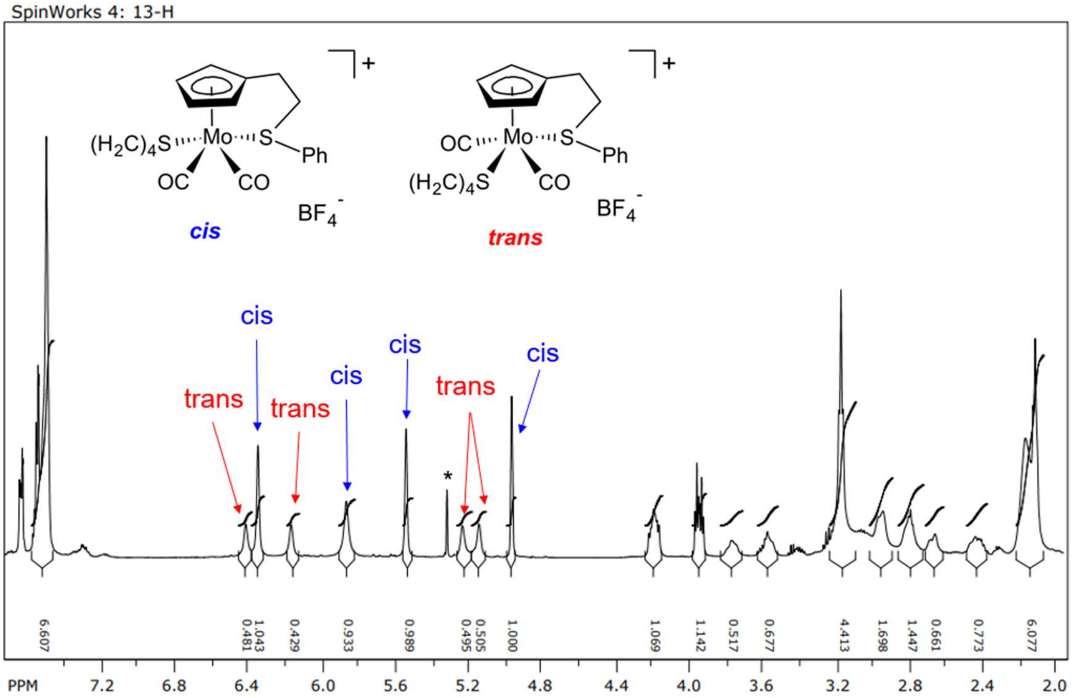

Figure S28: ^{1}H NMR spectrum of **13** in CD_2Cl_2 (500 MHz).

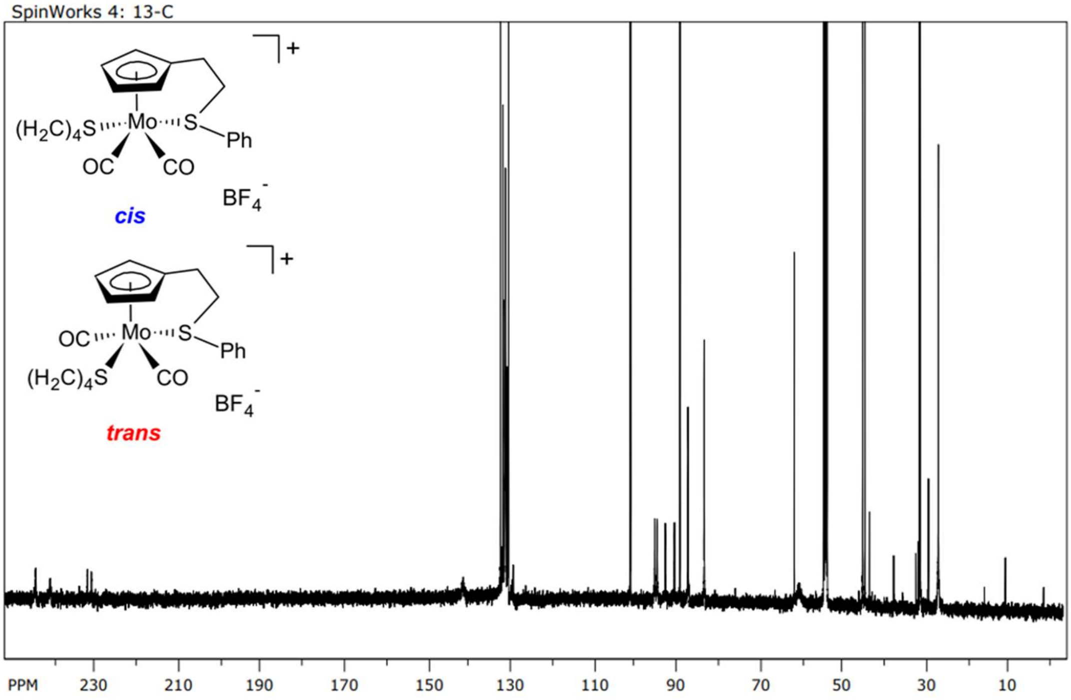

Figure S29: $^{13}C\{^{1}H\}$ NMR spectrum of **13** in CD_2Cl_2 (126 MHz).

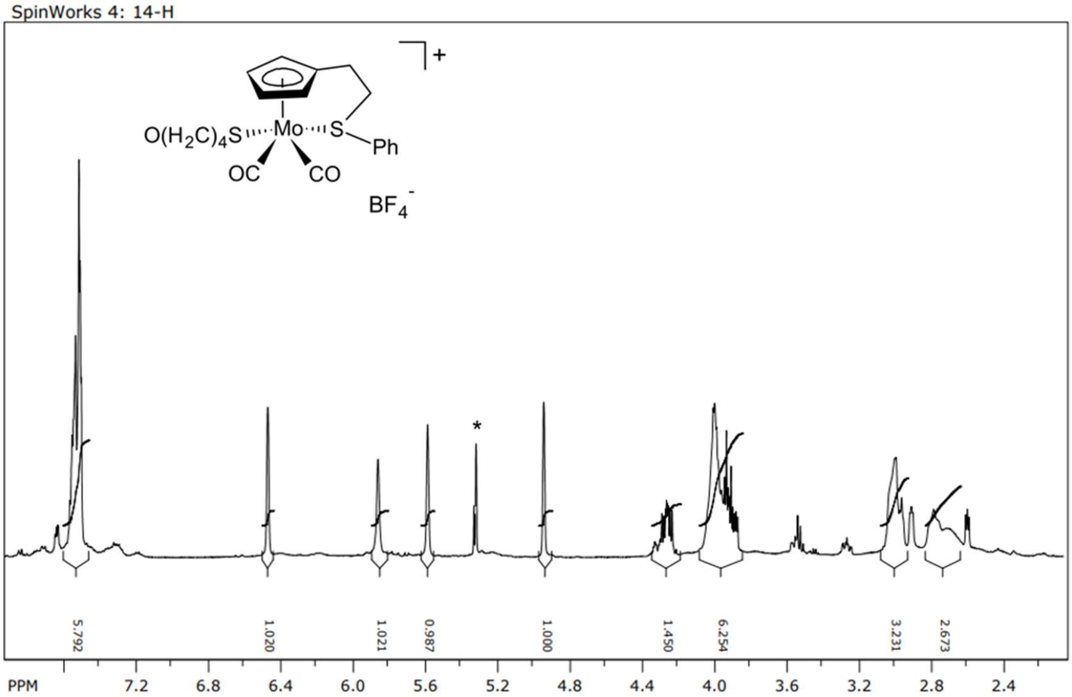

Figure S30: ^{1}H NMR spectrum of **14** in CD_2Cl_2 (500 MHz).

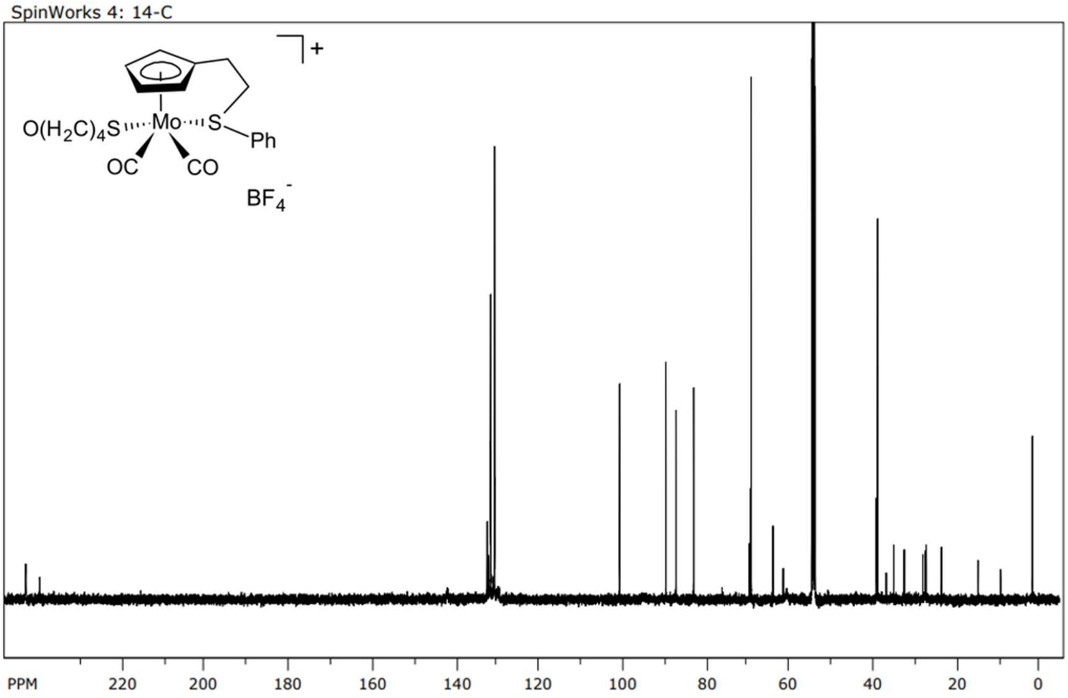

Figure S31: $^{13}C\{^{1}H\}$ NMR spectrum of **14** in CD_2Cl_2 (126 MHz).

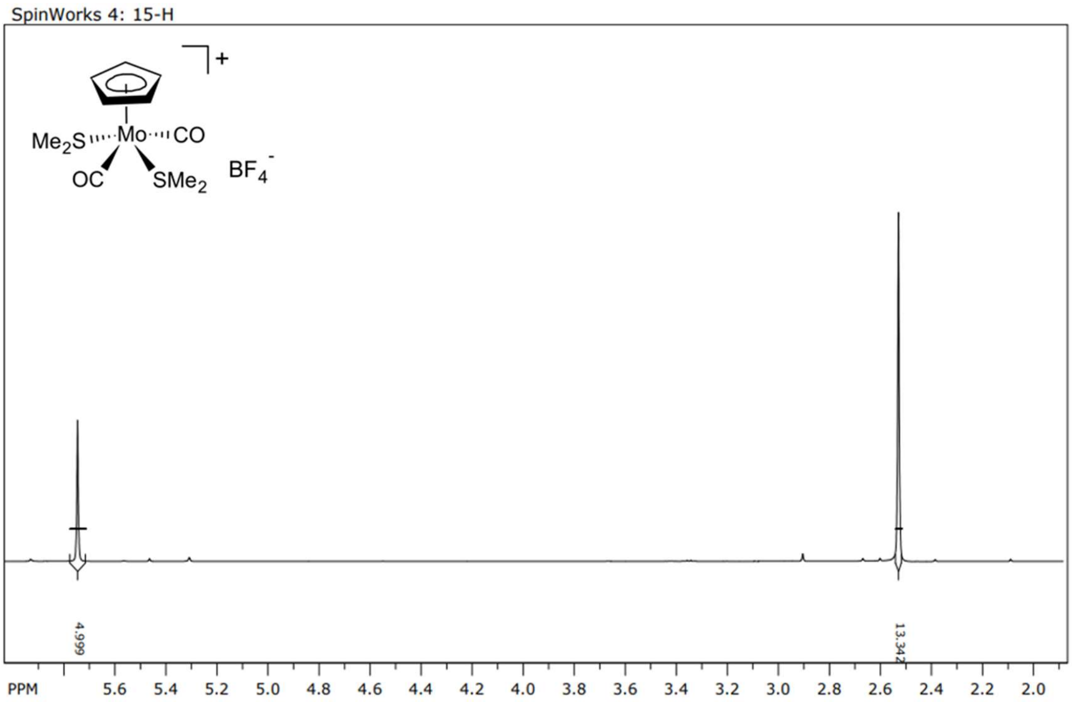

Figure S32: ^{1}H NMR spectrum of **15** in CD_2Cl_2 (500 MHz).

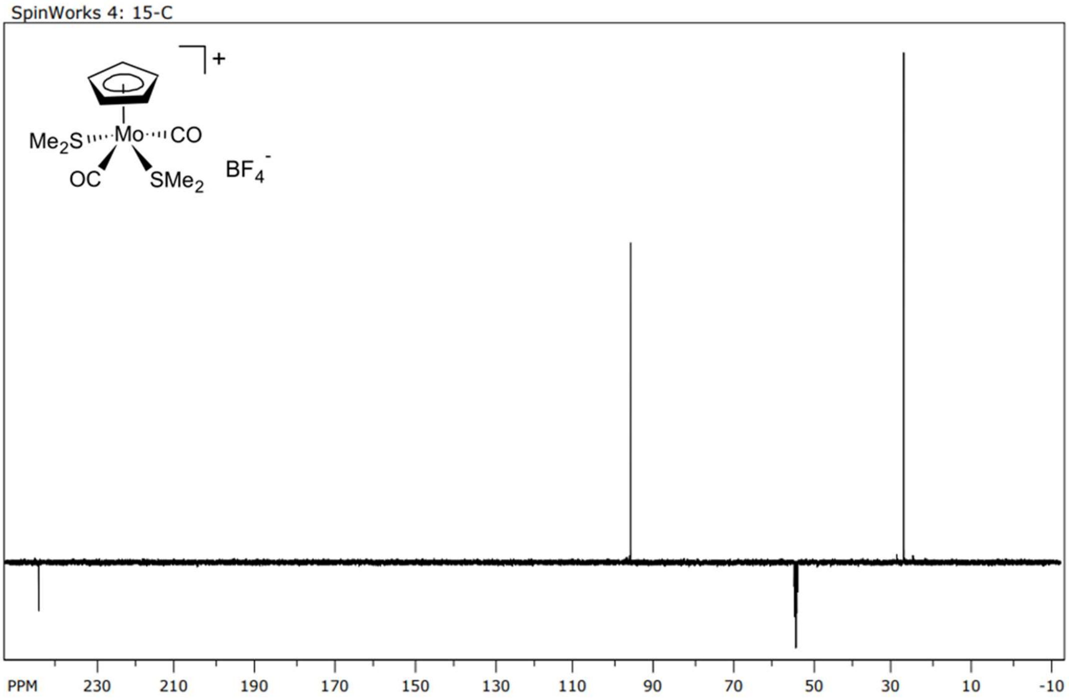

Figure S33: ^{13}C APT NMR spectrum of **15** in CD_2Cl_2 (126 MHz).

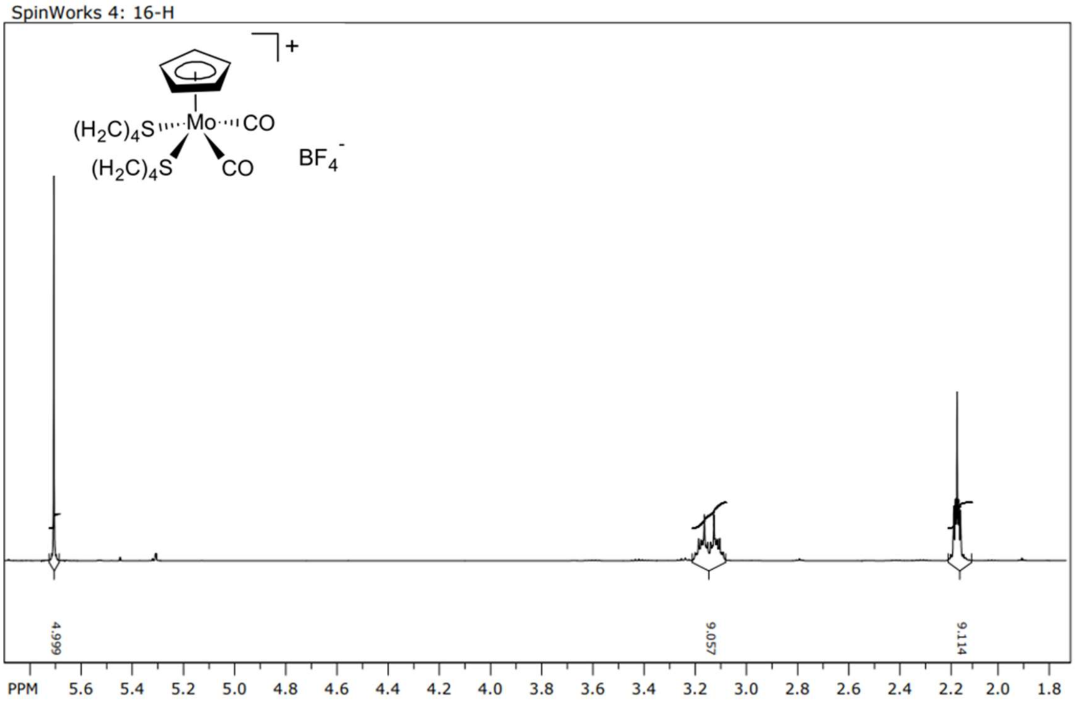

Figure S34: ^{1}H NMR spectrum of **16** in CD_2Cl_2 (500 MHz).

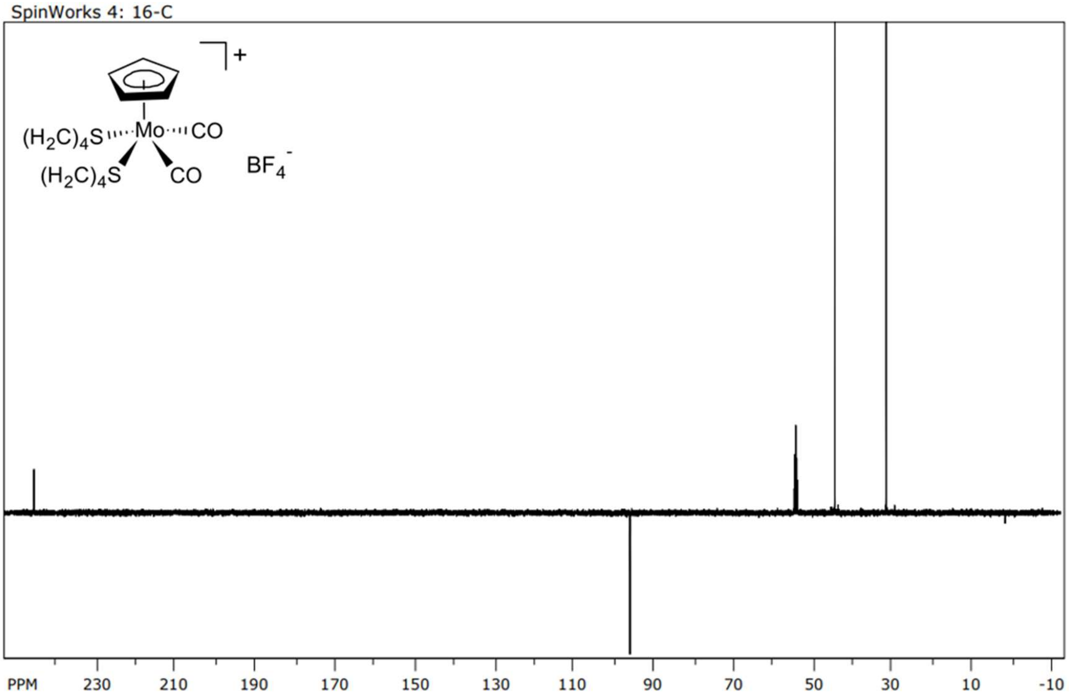

Figure S35: ^{13}C APT NMR spectrum of **16** in CD_2Cl_2 (126 MHz).

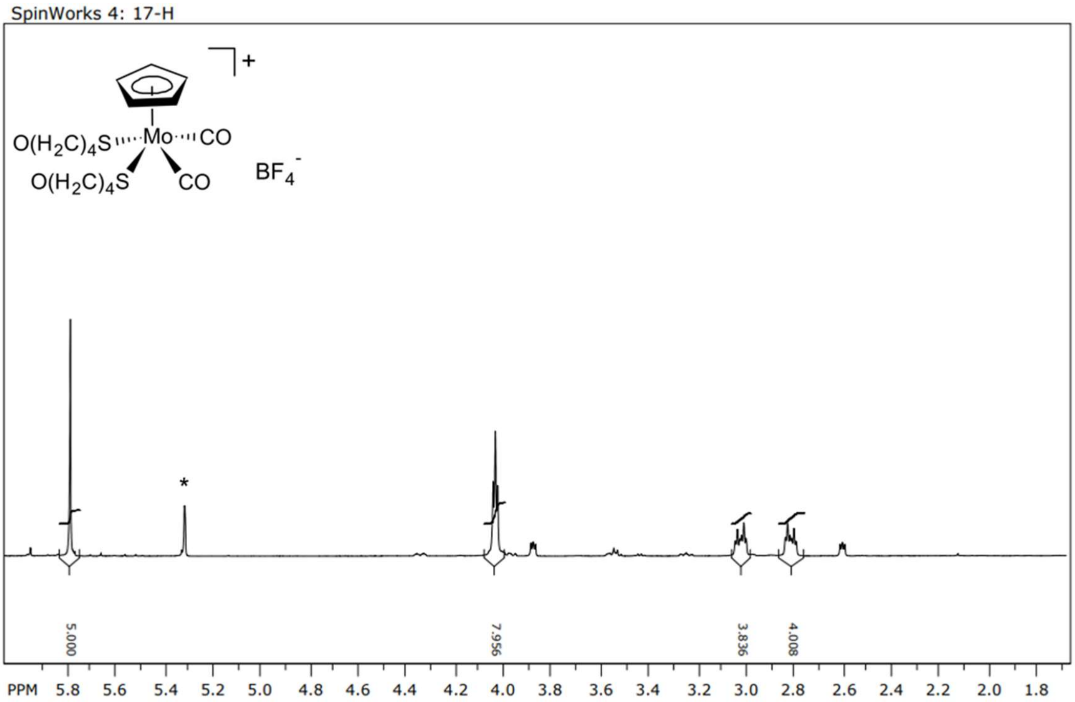

Figure S36: ^{1}H NMR spectrum of **17** in CD_2Cl_2 (500 MHz).

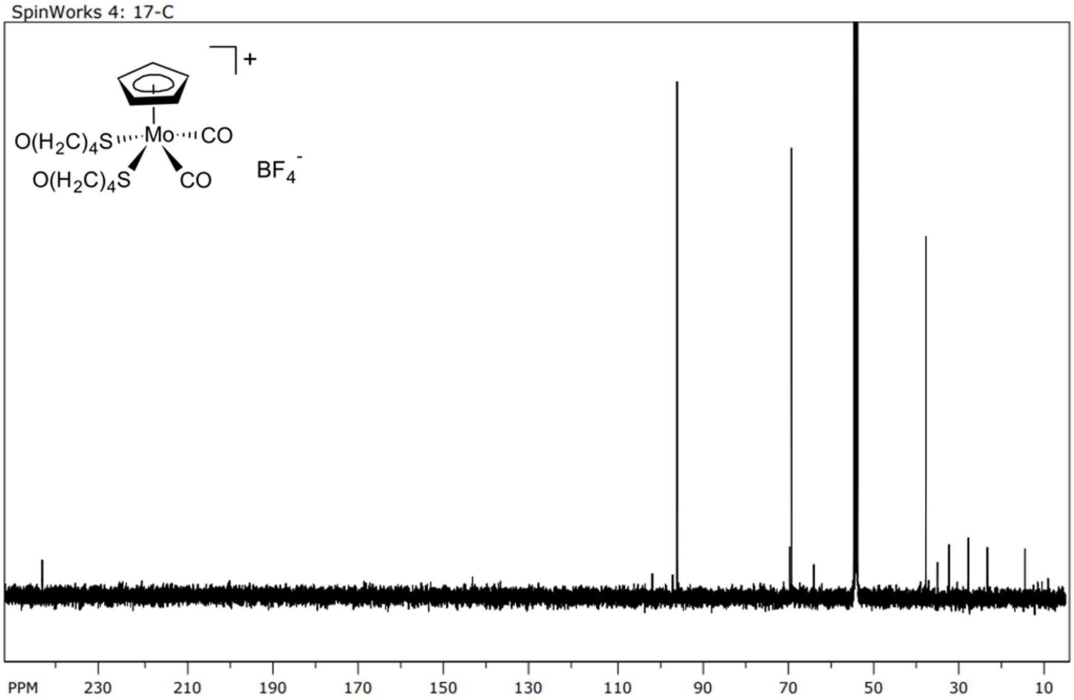

Figure S37: $^{13}C\{^{1}H\}$ NMR spectrum of **17** in CD_2Cl_2 (126 MHz).

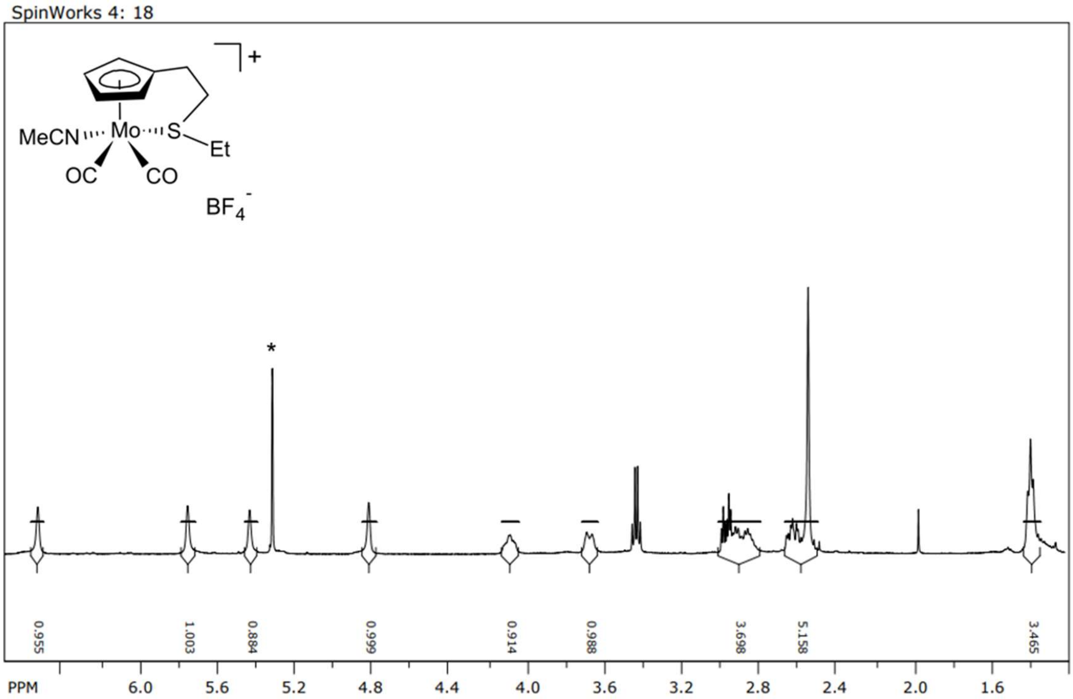

Figure S38: ^{1}H NMR spectrum of **18** in CD_2Cl_2 (500 MHz).

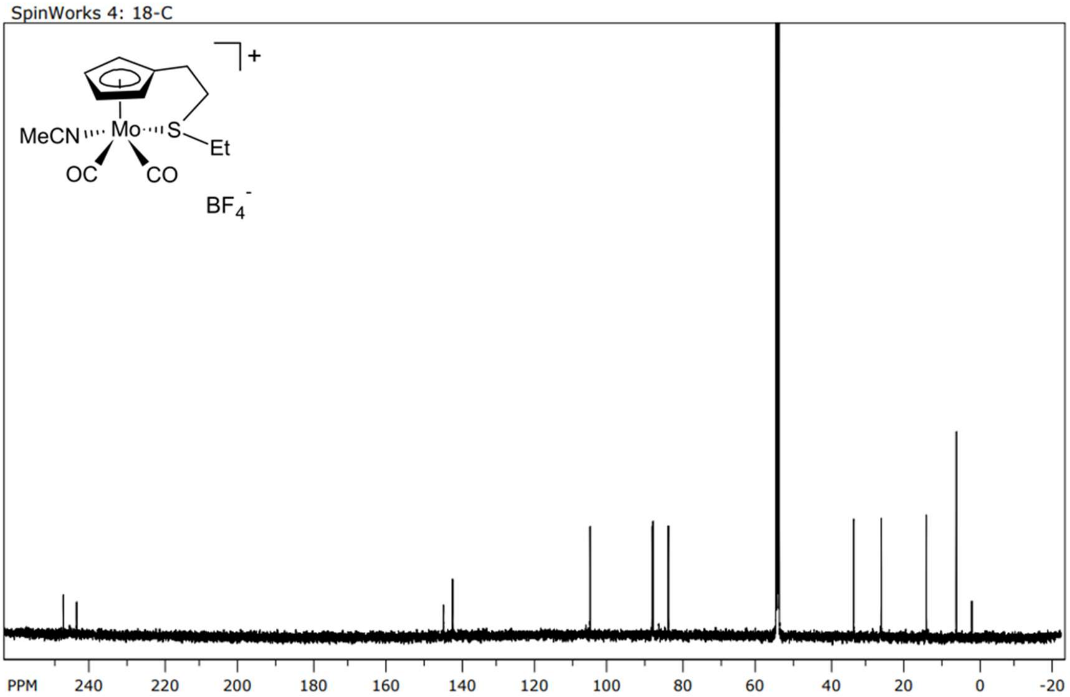

Figure S39: ^{13}C{^{1}H} NMR spectrum of **18** in CD_2Cl_2 (126 MHz).

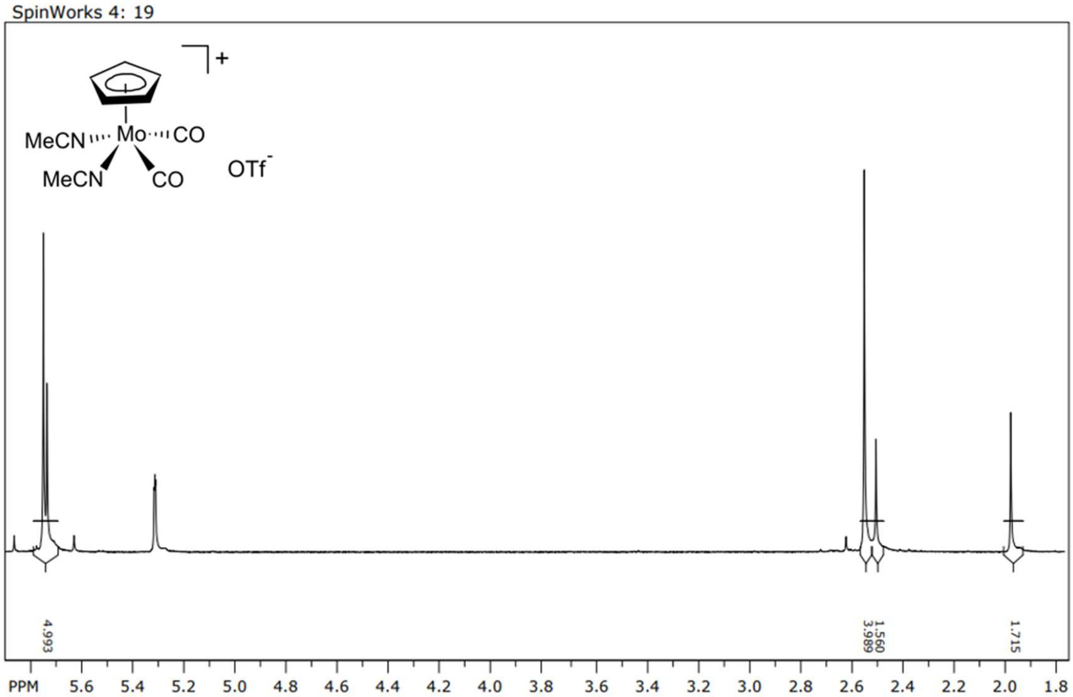

Figure S40: ^{1}H NMR spectrum of **19** in CD_2Cl_2 (400 MHz).
